# Identifying Effective Design Approaches to Allocate Genotypes in Two-Phase Designs: A Case Study in *Pelargonium zonale*

**DOI:** 10.3389/fpls.2017.02194

**Published:** 2018-01-05

**Authors:** Heike Molenaar, Robert Boehm, Hans-Peter Piepho

**Affiliations:** ^1^Biostatistics Unit, Institute of Crop Science, University of Hohenheim, Stuttgart, Germany; ^2^Klemm + Sohn GmbH & Co. KG, Stuttgart, Germany

**Keywords:** experimental design, two-phase design, mean variance of a pair-wise treatment difference, A-optimal, dummy analysis, experimental structure, horticultural breeding, *Pelargonium zonale*

## Abstract

Robust phenotypic data allow adequate statistical analysis and are crucial for any breeding purpose. Such data is obtained from experiments laid out to best control local variation. Additionally, experiments frequently involve two phases, each contributing environmental sources of variation. For example, in a former experiment we conducted to evaluate production related traits in *Pelargonium zonale*, there were two consecutive phases, each performed in a different greenhouse. Phase one involved the propagation of the breeding strains to obtain the stem cutting count, and phase two involved the assessment of root formation. The evaluation of the former study raised questions regarding options for improving the experimental layout: (i) Is there a disadvantage to using exactly the same design in both phases? (ii) Instead of generating a separate layout for each phase, can the design be optimized across both phases, such that the mean variance of a pair-wise treatment difference (MVD) can be decreased? To answer these questions, alternative approaches were explored to generate two-phase designs either in phase-wise order (Option 1) or across phases (Option 2). In Option 1 we considered the scenarios (i) using in both phases the same experimental design and (ii) randomizing each phase separately. In Option 2, we considered the scenarios (iii) generating a single design with eight replicates and splitting these among the two phases, (iv) separating the block structure across phases by dummy coding, and (v) design generation with optimal alignment of block units in the two phases. In both options, we considered the same or different block structures in each phase. The designs were evaluated by the MVD obtained by the intra-block analysis and the joint inter-block–intra-block analysis. The smallest MVD was most frequently obtained for designs generated across phases rather than for each phase separately, in particular when both phases of the design were separated with a single pseudo-level. The joint optimization ensured that treatment concurrences were equally balanced across pairs, one of the prerequisites for an efficient design. The proposed alternative approaches can be implemented with any model-based design packages with facilities to formulate linear models for treatment and block structures.

## Introduction

Robust phenotypic data from trials that allow an adequate statistical analysis are of utmost importance for successful varietal improvement, identification of quantitative loci, marker-assisted selection, association mapping, and genomic selection. To obtain such data, trials are laid out to best control local variability through an experimental design (Federer and Crossa, 2012). There are situations where the experiment consists of two phases, e.g., when plant material is grown in the field to obtain the yield in the first phase and in the second phase chemical analyses are conducted in the laboratory (Smith et al., 2014), in which case the environmental conditions in the field trial have an influence on the response obtained in the second phase of the experiment in the laboratory. In such situations, two-phase experimental designs are recommended. All too often, however, both the design and statistical analyses are less than optimal when the two-phase nature of the experiment is overlooked, e.g., the change of observational units from one phase to the other or an overlapping of phases (Brien et al., 2011). As a result, variation cannot be broken down into all its components, which leads to a decreased accuracy of treatment effect estimates (Curnow, 1959).

Two-phase experimental designs can be found in many research areas, for example in crop breeding programs, where plants are tested under field conditions during the first phase and collected material is processed further for chemical analysis; in clinical studies, where patients are treated first, and specimens are processed in a laboratory in the second phase; in food processing studies, when first mixtures are prepared and in a subsequent phase the mixtures are processed further to produce the final products (Brien et al., 2011); or when conducting microarray experiments, where first messenger RNA is derived from subjects that are exposed to a set of treatments and then the mRNA is used in a microarray assay to obtain the gene expression (Jarrett and Ruggiero, 2008). Even if a laboratory phase is not involved, two phases can be present, as in ornamental breeding, where in the first phase stock plants are cultivated and in the second phase harvested plant material is tested for production related traits (Molenaar et al., 2017). Both phases take place in greenhouses, which may be in different locations.

Often in planned two-phase experiments, the first phase is considered in the experimental design, while the second phase is not considered at all. For example, in cereal breeding, plant material from the field may be processed further according the “field order” resulting in a systematic allocation of treatments in the second phase or all samples of a treatment may be pooled together in the laboratory (Brien et al., 2011). Already in 1955, McIntyre (1955) described two-phase experimental designs and proposed the use of randomization in each phase.

Implementing a conventional two-phase design, where both phases use optimal designs, can pose some difficulties due to practical considerations as mentioned in a study on *Pelargonium zonale* by Molenaar et al. (2017). In that study, it would have been prohibitively labor-intensive to follow an optimized pre-defined layout, because of the elaborate process of planting thousands of stem cuttings in the second phase. As this was the first attempt to introduce a two-phase experimental design in a *P. zonale* breeding program, a compromise was made, and randomization in the second phase was carried out on site so that the requirement of randomization was met, but the design could not be optimized in view of the design used in the first phase.

This initial approach raised questions regarding options for further improving the experimental design: (i) Is there a disadvantage in leaving treatments in the same randomized order from the first phase when transferring samples to the second phase of the experiment, i.e., using exactly the same design in both phases? (ii) Instead of generating a separate layout for each phase, can the design be optimized across both phases, such that the MVD can be decreased across both phases compared to two independent designs?

Therefore, the objective of this study was to explore potential pragmatic approaches for generating improved two-phase experimental designs and thereby to answer questions (i) and (ii). Section “A Two-Phase Experiment in *P. zonale* Breeding” summarizes the former experiment of Molenaar et al. (2017), on which operational possibilities are modeled. Sections “Option 1 – Design Generation for Each Phase Separately” and “Option 2 – Design Generation across the Two Phases” present two options for generating two-phase experimental designs for each phase separately or across the two phases considering either the same or different block structures in both phases. In Section “Results”, the generated designs are evaluated regarding the MVD. Sections “Discussion and Conclusion”, give a discussion and conclusion to identify effective two-phase designs.

## Materials and Methods

### A Two-Phase Experiment in *P. zonale* Breeding

In 2013/14, we implemented a two-phase experimental design in a *P. zonale* breeding program to assess production related traits of *v* = 500 genotypes. In Phase 1 (P1), conducted in location 1, stock plants of genotypes were cultivated, from which the stem cutting count was obtained. In Phase 2 (P2), the stem cuttings harvested from genotypes during P1 were planted to assess the root formation in location 2. Both phases took place in greenhouses.

In P1, an α-design with *r* = 4 replicates, each with *b* = 167 incomplete blocks of size *k* = 3, was used. One of the incomplete blocks in each replicate was only of size two. Each experimental unit in P1 (EU1) contained a pair of stock plants from the same genotype, for a total of six plants per incomplete block of size three. Each cultivation table accommodated a full set of genotypes, i.e., one replicate.

**FIGURE 1:**
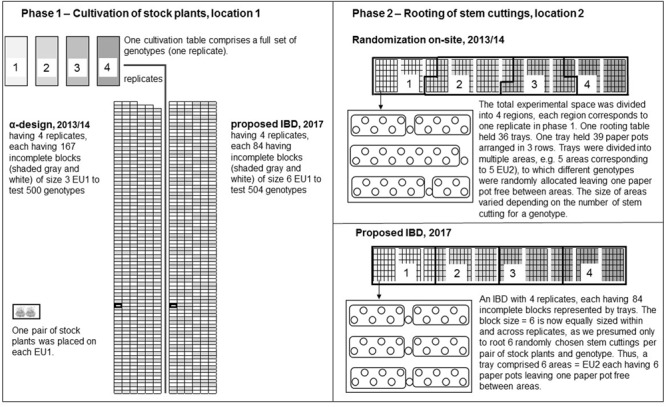
Modified according to Molenaar et al. (2017): Two-phase experimental design accounting for idealized conditions. In comparison to 2013/14, where an α-design with four replicates, each with 168 incomplete blocks of block size 3 (one of them was only of size 2), was used to test 500 genotypes in Phase 1 (P1). We idealized the conditions in such a way, that we could use instead an incomplete complete block design (α-design) with four replicates each having 84 incomplete blocks of size 6 to test 504 genotypes. In both cases, the cultivation tables comprised a complete set of genotypes placed on 500 and 504 planting positions, respectively. On each experimental unit, a pair of stock plants was placed. In Phase 2 (P2), when randomization was carried out on site in 2013/14, we divided the total experimental space of rooting tables into four regions to which the four replicates in P1 were systematically assigned. Regions shaded in gray in rooting tables in P2 correspond to replicates shaded in gray of cultivation tables in P1. Each rooting table held 36 trays and a tray contained 39 paper pots arranged in three rows. The trays were divided into areas comprising different numbers of paper pots to which genotypes were randomly allocated. The number of paper pots, i.e., the size of areas (experimental units in P2), varied depending on the numbers of stem cuttings for a genotype. In accordance with our idealized conditions assuming that only six stem cuttings of a pair of stock plants and genotype are rooted, we proposed to use a pre-specified IBD with four replicates each having 84 incomplete blocks of size 6 in P2. Thus, the regions, to which the replicates in P1 were assigned, the incomplete blocks represented by the trays and the EU2 were now of equal size.

In P2, randomization was carried out on site as follows: First, the total experimental space of rooting tables was divided into four regions. To each region in P2 the replicates of P1 were systematically assigned. A rooting table held 36 trays and a region held 72 up to 108 trays, hence, not all trays fit necessarily on one rooting table. A tray held 39 paper pots arranged in three rows. Trays were divided into areas representing EU2. Second, all stem cuttings of a genotype and replicate in P1 were packed in a small bag to transfer the plant material from location 1 to 2, and were randomly allocated to one area in the corresponding region to which a replicate was assigned in P2. Thus, stem cuttings from each EU1 were allocated exactly to one EU2. The sizes of areas for EU2 varied depending on the harvested stem cutting count per genotypes. The rooting tables and trays in P2 were considered as *post-blocking* factors in the analysis, which could be regarded as incomplete blocks in an IBD, for which the design was previously not optimized (**Figure 1**).

### Idealized Conditions to Assume the Same Block Structure in Both Phases

We idealized the experimental conditions in several ways to different degrees in the designs to be described in this and the following Sub-section “Different Block Structures in Both Phases” for comparing different design generation scenarios. This was done in the interest of focusing on the general principles implemented in scenarios investigated in this study without having to focus on intricate specifics of the *P. zonale* study. First, we assumed that in each phase the same block structure can be used. Thus, for each pair of stock plants we presumed that no stock plants were lost and that the stem cutting counts were reduced to six stem cuttings per genotype in P1 to assess root formation in P2. Hence, the EU2 (areas) were assumed to be of equal size in P2.

Further, the physical unit of a tray should correspond exactly to one randomization unit in P2, i.e., to an incomplete block of size six. To consider the same block structure in both phases so that block units in P1 correspond to block units of the same size in P2, requires increasing the block size in P1 from *k* = 3 to *k* = 6. These idealized conditions enabled us to assume the same resolvable IBD design with *r* = 4 replicates with each *b* = 84 incomplete blocks having the same block sizes *k* = 6 in both phases (**Figure 1**). We also assumed equal block sizes and the genotype number was increased from *v* = 500 to 504. Given these design properties, two options were considered to generate the two-phase experimental design (**Table 1**): Option 1 was to generate a design for each phase separately (Section “Option 1 – Design Generation for Each Phase Separately”) and Option 2 was to generate a design across phases by simultaneously accounting for the block structure of both phases (Section “Option 2 – Design Generation across the Two Phases”). During design generation using either Option 1 or Option 2, the complete replicates from P1 were kept intact in P2, except for *Scenario III*.

**Table 1:** Overview of designs with the same block structure in both phases^†^.

Option	Description	Scenario	Code in Supplementary Presentation 1	Figure in Supplementary Presentation 2
1 – Design generation separately for each phase	Exactly the same design in both phases	*I*	1	A
	New randomization of genotypes to IB within replicates of P2	*II*	2	B
2 – Design generation across the two phases	Generating a single design with eight replicates and splitting these among the two phases	*III*	3	C
	Separation of block structures using phase-specific dummy coding	*IV*	4	D
	Design generation in two steps: (i) allocating blocks of P2 to incomplete blocks of P1; (ii) allocating of genotypes to IB of both P1 and P2	*V*	5	E1 and E2

*^†^In each phase, an incomplete block design was used with *v* = 504 genotypes, *r* = 4 replicates, *b* = 84 incomplete blocks of size *k* = 6. Designs were generated for each phase separately or across phases and for different scenarios within these options.*

#### General Approach

The general approach for generating two-phase experimental designs by either Options 1 or 2 was model-based. Therefore, first a treatment model was defined and second a block model for each of the two phases. Such model-based approaches can be implemented in various software packages, e.g., *dae* (Brien, 2017), *DiGGer* (Coombes, 2009), or *OD* (Butler, 2013) in R or the OPTEX procedure in SAS (SAS Institute Inc., 2014). We implemented our approaches with OPTEX, and provided all relevant codes (**Supplementary Presentation 1**).

#### Option 1 – Design Generation for Each Phase Separately

##### Scenario I – Transmitting the experimental layout of P1 to P2

A resolvable IBD was generated for P1 for the specifications given above (Code 1, Figure A in **Supplementary Presentation 2**). The experimental layout of P1 was transmitted exactly to P2. In doing so, treatments in P2 were left in the same order as used in P1.

##### Scenario II – New randomization of genotypes to incomplete blocks within replicates in P2

In contrast to *Scenario I*, in P2 a separate resolvable IBD was generated (Code 2, Figure B).

#### Option 2 – Design Generation across the Two Phases

##### Scenario III – Generating a single design with eight replicates and splitting these among the two phases

We generated a resolvable IBD with *r* = 8 replicates (Code 3, Figure C), where all other design parameters remained unchanged (*v =* 504, *b =* 84, *k =* 6), and split these replicates equally among the two phases. Since genotypes were randomized to incomplete blocks across the eight replicates, the design was optimized across the two phases in terms of the number of concurrences per treatment pair. Each complete replicate in P1 was transferred intact to one replicate in P2.

##### Scenario IV – Separation of block structures using phase-specific dummy coding

In the dataset defining the block structures across phases, we defined a factor identifying the two phases (Code 4, Figure D). In each phase, there were *r* = 4 replicates and incomplete blocks were nested within each of the replicates. The records for the two phases were concatenated in the dataset for design generation based on a model comprising the block effects for both phases. The clue for generating the design across the two phases was to set the factor for incomplete blocks of P1 to a single pseudo level for incomplete blocks of P2 and vice versa. By this dummy coding, the pseudo level acted as one additional block level of incomplete blocks in P1 or P2. The design was then optimized simultaneously with respect to the assignment of genotypes to the two blocking systems.

##### Scenario V – Design generation with optimal alignment of block units in the two phases

Instead of generating the design in phase-wise order, the design generation was conducted in replicate-wise order in two steps (Code 5, Figures E1, E2), optimizing the alignment of block structures of both phases. First, for each replicate, the incomplete blocks of P2 were allocated to incomplete blocks of P1 for each of the four replicates separately to obtain a block layout of the design across the two phases. This was achieved by formally considering blocks of P2 as the “treatment” factor and blocks in P1 as the “block” factor, thus optimizing the efficiency of block effects estimates in P2, given the block structure in P1. In this step, the allocation of genotypes to EU1 and EU2 was not yet considered. Second, the genotypes were allocated to incomplete blocks within replicates of both P1 and P2 considering all four replicates simultaneously. At this stage, the alignment of blocks in P1 with blocks in P2 was fixed at the configuration obtained in the first step, and this alignment was used as the block model to generate an allocation of treatments to EU1 and EU2 simultaneously.

### Different Block Structures in Both Phases

The assumption of a common block structure in both phases is rather idealized as in practice the phases of the experiments take place in totally different locations and different environmental conditions prevail in each phase (Molenaar et al., 2017). To account better for the environmental conditions in location 1, two different *post*-*blocking* factors were considered in the former analysis, which are now further considered in generating designs.

#### Adding a Column Factor in the First Phase

A column factor was added to the *randomization*-based model, representing columns of 84 experimental units per replicate on tables in the greenhouse. Accommodating this column factor means that different block structures are used in both phases. Hence, the operational approaches derived in Section “Option 1 – Design Generation for Each Phase Separately” were modified, using in P1 a row-column design to test *v* = 504 genotypes in *r* = 4 replicates, each arranged in *k* = 84 rows and *s* = 6 columns, whereas in P2 the resolvable IBD used previously was employed (**Table 2**).

**Table 2:** Overview of designs assuming different block structure in each phase^†^ for the same two options as in **Table 1**.

Option	Design in		Description	Scenario	Code in Supplementary Presentation 1
	Phase 1	Phase 2			
1 - Design generation for each phase separately	Row–column design	Resolvable IBD		*VI*	6
	Row–column design considering the ”*worker-day*”	Resolvable IBD		*VII a, b, c*	7
	Considering only the “*worker-day*” within replicates	Resolvable IBD		*VIII a, b, c*	8

2 - Design generation across phases	Row–column design	Resolvable IBD	Separation of block structures using phase-specific dummy coding	*IX*	9
			Design generation in two steps: (i) allocating blocks of P2 to rows and columns of P1; (ii) allocating genotypes to rows and columns of P1 and IB of P2	*X*	10
	Row–column design considering the ”*worker-day*”	Resolvable IBD	Separation of block structures using phase-specific dummy coding	*XI a, b, c*	11
			Design generation in two steps: (i) allocating blocks of P2 to rows, columns and “*worker-day*” of P1; (ii) allocating genotypes to rows, columns and “*worker*-*day*” of P1 and IB of P2	*XII a, b, c*	12
	Considering only the “*worker-day*” within replicates	Resolvable IBD	Separation of block structures using phase-specific dummy coding	*XIII a, b, c*	13
			Design generation in two steps: (i) allocating blocks of P2 to “*worker-day*” of P1; (ii) allocating genotypes to the “*worker*-*day*” of P1 and IB of P2	*XIV a, b, c*	14

*^†^In phase 1, a row-column design with *v* = 504 genotypes, *r* = 4 replicates, *z* = 84 rows and *s* = 6 columns and in phase 2 a resolvable IBD with *r* = 4 replicates, *b* = 84 incomplete blocks of size *k* = 6 were assumed.*

#### Blocking Factor to Account for Induced Variations by Workers in the First Phase

**FIGURE 2:**
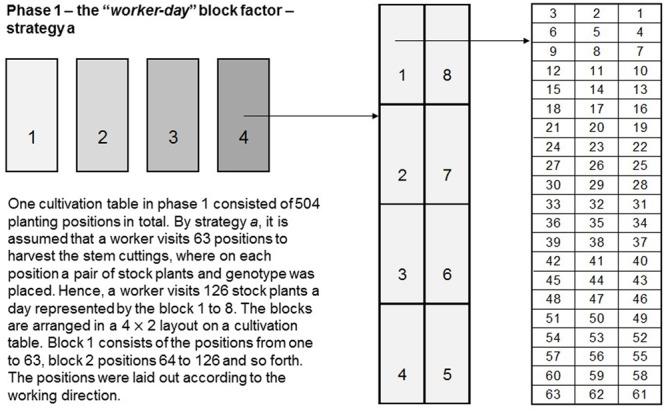
Schematic representation of the “*worker-day*” blocking strategy *a*. One replicate in P1 consisted of 504 planting positions. On each position a pair of stock plants of a genotype was placed. By blocking strategy *a*, it is assumed that a worker visits 63 positions to harvest the stem cuttings of 126 stock plants a day represented by the blocks 1 to 8, which are arranged in a 4 × 2 layout on a cultivation table.

Molenaar et al. (2017) found that in most cases the largest variance was the residual error variance, while in P1 the incomplete block variance was estimated to be zero, indicating that there was no correction due to that block factor during the estimation of effects when modeled as random. In search of sources of variation that were not explicitly taken into account so far, we found that workers harvesting the stem cuttings in P1 induced some effect. Considering that a worker can harvest stem cuttings from approximately 125 stock plants per day, an additional *post-blocking* factor “*worker-day*” was defined, which comprised eight levels (blocking strategy *a*). Levels one to eight corresponded to the positional numbers from 1 to 63, 64 to 126, 127 to 189, 190 to 252, 253 to 315, 316 to 378, 379 to 441 and 442 to 500 in the layout of EU1 in each replicate (**Figure 2**). However, during cultivation within the first 5 months stock plants were lost at random. Hence, less than 125 stock plants per “*worker-day*” were grouped together for analysis. Therefore, two other block strategies (*b* and *c*) in terms of the number of planting positions visited by a worker per day were defined. We further considered this additional block factor within a row–column design, but also as the only block factor in P1, for generating designs using either Options 1 or 2 (**Table 2**).

### The Mean Variance of a Treatment Difference as a Selection Criterion

Designs generated by the procedure OPTEX are optimized for D-efficiency (*OD* or *DiGGer* packages provide algorithms for generating A-optimal designs). In plant breeding A-optimal or A-efficient designs are preferred as optimizing this criterion minimizes the average variance of genotype differences (MVD) (Hinkelmann and Kempthorne, 2005). Thus, the precision of estimates of genotype differences is increased and better phenotypic selection and varietal improvement can be achieved. Both D- and A-optimality usually lead to similar designs for comparative experiments with a single treatment factor (John and Williams, 1995), so the procedure OPTEX was a useful tool for our purposes, despite its focus on D-optimality. Thus, we computed the MVD obtained by linear mixed models either by intra-block or joint inter-block-intra-block dummy analyses (Piepho, 2015), in which the information about the precision of genotype parameters is contained in the variance-covariance matrix (Mead, 1988). Generally, the design showing the lowest MVD was preferred.

#### Resolvability

We ensured that all designs were resolvable, meaning that the *b* incomplete blocks containing *k* plots (EUs) can be grouped to a complete *r* replicate of the *v* treatments. Resolvability of all designs generated was verified by frequency tables for the genotype-by-replicate classifications in each phase. For a resolvable IBD, all entries in the table must be unity. If necessary, resolvability of the two-phase designs was enforced by defining effects for incomplete blocks as random effects, tuning the variance so that resolvability was achieved. Defining a block effect as random essentially allows tuning its influence on the treatment information matrix. The smaller the variance, the smaller the influence on the treatment information matrix. In OPTEX, the variance of an effect is tuned via the PRIOR option (see Pereira and Tobias, 2015, for more details). For design generation in each scenario we set the prior for replicates to zero. For all remaining block effects, the prior was set to 2016, the value corresponded to the total number of experimental units in the experiment (504 × 4 = 2016 EU1 = EU2). If resolvability was not achieved the prior was increased until resolvability was achieved.

#### Model Set-up for Design Evaluation

As mentioned above, our approach generally requires specification of the treatment model on the one hand and the model for block effects on the other hand. The model notation used here is universally applicable in any design package allowing the specification of linear models.

We illustrate this general model set-up for either Options 1 or 2 by considering a two-phase design having the same block structure in both phases. The treatment model, representing the ‘randomized-tier’ (Brien and Demétrio, 2009), was

(1) $$\begin{array}{c} \mathrm{GEN}, \end{array}$$

where GEN denotes the genotypes.

When the designs were generated for each phase separately (Option 1), then the *randomization*-based block model for design effects was set up for each phase separately. The P1 block model was

(2) $$\begin{array}{c} REP+REP.IB1+\underline{\mathrm{REP}.IB1.\mathrm{PAIR}} \end{array}$$

and the *randomization*-based model for P2 was

(3) $$\begin{array}{c} REP+REP.IB2+\underline{\mathrm{REP}.IB2.\mathrm{AREA}} \end{array}$$

where REP denotes the replicates, REP.IB1, the incomplete blocks nested within replicates in P1, REP.IB1.PAIR, the residual error in P1, i.e., the EU1, on which a pair of stock plants was placed and represented the observational unit. Further, REP.IB2 denotes the incomplete blocks within replicates in P2 and REP.IB2.AREA, the residual error in P2, i.e., the EU2, from which the root formation of stem cutting was assessed. It is noted, that in model (2) and (3), REP was considered as a fixed effect, whereas incomplete blocks and the residual error were considered as random effects for generating designs.

The full model for design evaluation was obtained by augmenting the treatment model with both phase-specific *randomization*-based block models,

(4) $$\begin{array}{c} GEN+REP+REP.IB1+REP.IB2+\underline{\mathrm{REP}.IB2.\mathrm{AREA}} \end{array}$$

As the replicates were kept intact from P1 to P2, only one effect was needed to define the replicates. Defining the experimental unit of the full model, the EU1 effect REP.IB1.PAIR did not need to be added explicitly either as it was implicitly accounted for by the EU2 effect REP.IB2.AREAS. This is because one EU1 was allocated to one EU2 and hence, effects of EU1 and EU2 are confounded.

When designs were generated across phases (Option 2), the block model for design generation was

(5) $$\begin{array}{c} REP+REP.IB1+REP.IB2+\underline{\mathrm{REP}.IB2.\mathrm{AREA}}\;\left(5\right) \end{array}$$

The full model corresponds to model (4), except for *Scenario III*, where the design was generated across the two phases by increasing the replicate number from four to eight. The model for design generation in this case was model (1), but after splitting the eight replicates among the two phases and recoding the incomplete blocks for P1 and P2 as IB1 and IB2, respectively, the model for analysis was model (4).

Considering different block structures in both phases, e.g., in P1 a row-column design, a row-column design with the additional block factor “*worker-day*” or only the “*worker-day*” and in P2 still utilizing a resolvable IBD, the block factor of P1 (IB1) in models (2) and (4) was replaced by rows nested within replicates (REP.ROW), columns nested within replicates (REP.COL) and “*worker-day*” (REP.WORK), respectively (**Table 3**).

**Table 3:** Full models in *Scenarios I* to *XIV*, including treatment effects, design effects in Phase 1 (P1) and Phase 2 (P2) and error terms used to estimate MVD for design evaluation.

Scenario	Model^‡^	Treatment effect	Design effects^§^					ERROR
			P1				P2	
*I*	2	GEN	REP	REP.IB1				REP.IB1.PAIR
*II–V*	4	GEN	REP	REP.IB1			REP.IB2	REP.IB2.AREA
*VI, IX, X*	5	GEN	REP	REP.ROW	REP.COL		REP.IB2	REP.IB2.AREA
*VII, XI, XII*^†^	6	GEN	REP	REP.ROW	REP.COL	REP.WORK	REP.IB2	REP.IB2.AREA
*VIII, XIII, XIV*^†^	7	GEN	REP			REP.WORK	REP.IB2	REP.IB2.AREA

*^†^For each scenario all blocking strategies a–c of “worker-day” were considered.*

*^‡^Model (2): IBD, which was transmitted from P1 to P2; Model (4): IBD in P1, IBD in P2; Model (5): IBD in P1 with the additional *post-blocking* factor column, IBD in P2; Model (6) a–c: IBD with the additional *post-blocking* factor column and “*worker-day*”, where a to c indicate the different blocking strategies to account for the different numbers of positions that can be met by one worker per day. Model (7) a–c: Considering only the “worker-day” block in P1, and an IBD in P2.*

*^§^REP is the replicate effect, REP.WORK is the “*worker-day*” effect, REP.IB1 is the incomplete block effect in the first phase, REP.ROW is the row effect in the first phase, REP.COL is the column effect in the first phase, REP.IB2 is the incomplete block effect in the second phase, ERROR is the residual error.*

#### The Estimation of Variance Components (VC)

Variance components (VC) were required for the dummy analyses in the next step in Section “Intra-block or Joint Inter-block–Intra-block Dummy Analysis”. Hence, for each block effect, including the residual error, the VC were estimated from Models (4) to (7) (**Table 3**) and the experimental data 2013/14 by taking all block effects as random. Because of the idealized conditions, IB2 was equivalent to the *post-blocking* factor TRAY in P2, whereas the other *post-blocking* factor TABLE in P2 of the past analysis (Molenaar et al., 2017) was neglected in the current analysis, because of confounding with the replicate effect (REP).

#### Intra-block or Joint Inter-block–Intra-block Dummy Analysis

The MVD was obtained from models (2) to (7) by a dummy analysis (**Supplementary Presentation 3**) taking block effect either as fixed or as random for each two-phase designs implemented in each scenario. The analysis with fixed blocks utilized the intra-block information and the obtained MVD_(F)_ depended only on the residual error variance (fixed at the value of residual error variance obtained in the previous experiment) and the design, whereas random blocks allowed also the recovery the inter-block information (John and Williams, 1995). The variance of random block effects was set to estimated VC (**Table 4**) to obtain the MVD_(R)_. Now, the MVD_(R)_ depended not only on the residual error variance and the design, but also on the block variances estimated from the previous experiment. Further, the MVD was also obtained from the previous experiment applying the same models for the intra- and the joint inter-block–intra-block analysis to illustrate on the one hand the gain in precision by using the two *post-blocking* factors column and “*worker-day*” in the first phase, and, on the other hand, to compare the precision of the former experiment with its improved modifications implemented in each scenario. In analyzing *Scenario I*, the VC of IB1 and IB2 estimated by the use of model (4) (**Table 4**) were summarized and assigned to model (2) with VC of the replicate effect and residual error, estimated from model (4) too, to obtain the MVD_(R)_.

**Table 4:** Variance components of each model effect and corresponding proportions of the total variation attributable to each effect for Models (4) to (7).

	Model effect^†^	Model^‡^					
		4	5	6, *a*	6, *b*	6, *c*	7, *a*	7, *b*	7, *c*
	REP	2.6469	2.6378	2.6406	2.5939	2.5796	2.6192	2.5743	2.5621
	REP.WORK	–	–	0.2569	0.3204	0.3097	0.3131	0.3542	0.3352
	REP.IB1	0.1303	–	–	–	–	–	–	–
	REP.ROW	–	0.0505	0.0443	0.0418	0.0172	–	–	–
	REP.COL	–	0.2043	0.0823	0.0568	0.0497	–	–	–
	REP.IB2	0.5066	0.4378	0.4518	0.4516	0.4616	0.4892	0.4780	0.4854
	ERROR	3.7806	3.7315	3.5895	3.5767	3.6381	3.6524	3.6360	3.6729
	
Sum VC		7.0744	7.0619	7.0654	7.0412	7.0559	7.0739	7.0425	7.0556

	REP	37.5565	37.3526	37.3738	36.8388	36.5595	37.0263	36.5538	36.3130
	REP.WORK	–	–	3.6360	4.5503	4.3892	4.4261	5.0295	4.7508
	REP.IB1	1.8419	–	–	–	–	–	–	–
Proportion in %	REP.ROW	–	0.7151	0.6270	0.5934	0.2442	–	–	–
	REP.COL	–	2.8930	1.1644	0.8072	0.7039	–	–	–
	REP.IB2	7.1610	6.1995	6.3946	6.4137	6.5420	6.9156	6.7874	6.8796
	ERROR	53.4406	52.8399	50.8041	50.7966	51.5611	51.6321	51.6294	52.0565
Total		100%	100%	100%	100%	100%	100%	100%	100%

*^†^REP is the replicate effect, REP.WORK is the “*worker-day*” effect, REP.IB1 is the incomplete block effect in the first phase, REP.ROW is the row effect in the first phase, REP.COL is the column effect in the first phase, REP.IB2 is the incomplete block effect in the second phase, ERROR is the residual error.*

*^‡^Model (4): IBD in P1, IBD in P2; Model (5): IBD in P1 with the additional *post-blocking* factor column, IBD in P2; Model (6) a–c: IBD with the additional *post-blocking* factor column and “*worker-day*”, where *a* to *c* indicate the different blocking strategies to account for the different numbers of positions that can be met by one worker per day. Model (7) a–c: considering only the “*worker-day*” block in P1, and an IBD in P2.*

## Results

### Resolvability

In all scenarios resolvability was achieved by setting the prior value for incomplete block effects in the block model specification to the total number of experimental units, 2016, except for *Scenarios V, X, XII a–c* and *XIV a–c*. Resolvability was realized for those scenarios by increasing the prior value to 10^6^.

### VCs of Random Effects

The largest VC was the residual error variance with a proportion of 53.44% of the total variance, followed by the replicate effect with 37.56 % (**Table 4**). By comparison, the variance of the incomplete block effect in P1 was small (1.8%). After adding the column *post-blocking* factor to the first phase and still using a resolvable IBD in P2 [model (5)], the residual error variance could be reduced from 53.5 to 52.8%. The proportion of variation explained by the rows was below 1%, whereas the proportion of variation explained by columns was 2.8%. The residual error variance was further reduced by accounting for the block factor “*worker-day*” in P1 to 50.8%, where simultaneously the proportion of variation explained by row and column effects was reduced to below 1% [model (6) *a–c*]. Subsequently, scenarios were considered, where the “*worker-day*” was the only block factor in P1 and the resolvable IBD was retained in P2 [model (7) *a–c*]. By doing this, the proportion of variation explained by the replicate effects was retained, the proportion of variation explained by the “*worker-day*” effect in P1, and the proportion of variation explained by the incomplete block effect in P2, were maximized. However, the proportion of variation captured by the residual error was increased again by 1% compared to model (6) *a–c*.

### The Precision of the Two-Phase Design in 2013/14

In *post hoc* analysis of the previous experiment, the greatest MVD_(F)_ and MVD_(R)_ were observed generally in all conducted dummy analyses for model (4) (**Table 5**). By adding a column factor in the first phase [model (5)], a reduction of MVD_(F)_ was achieved by more than 50%, whereas the reduction in MVD_(R)_ was rather small. By considering the “*worker-day*” factor [model (6) *a*–*c*] in addition to the column factor, the reduction in MVD was about 0.04. When only the “*worker-day*” factor in the first phase [models (7), *a*–*c*] was considered, the reduction in MVD_(F)_ was below 3.0, whereas the MVD_(R)_ was slightly higher than the MVD_(R)_ obtained by models (6), *a*–*c* (**Table 5**).

**Table 5:** Different models for evaluating MVD for two additional blocking factors where MVD is obtained either by assuming blocks to be fixed or random.

Model^†^	MVD_(F)_	MVD_(R)_
4	9.4238	2.6767
5	4.55164	2.6147
6, *a*	4.51763	2.5499
6, *b*	4.41603	2.5270
6, *c*	4.51641	2.5399
7, *a*	2.78311	2.5541
7, *b*	2.74511	2.5303
7, *c*	2.74876	2.5425

*^†^Model (4): IBD in P1, IBD in P2; Model (5): IBD in P1 with the additional *post-blocking* factor column, IBD in P2; Model (6) *a*–*c*: IBD with the additional *post-blocking* factor column and “*worker-day*”, where a to c indicate the different blocking strategies to account for the different numbers of position that can be met by a worker and a day; Model (7) *a*–*c*: considering only the “worker-day” block in P1 and an IBD in P2.*

### The Precision of Alternative Approaches

#### Two-Phase Designs Containing the Same Block Structure in Both Phases

By using the same pre-defined design in both phases, the smallest MVD_(F)_ and MVD_(R)_ were obtained for *Scenario I* (experimental layout was transmitted from P1 to P2) (**Table 6**). The MVD_(R)_ were quite similar, especially between *Scenario II* to *IV*, whereas values of MVD_(F)_ showed a wider range (2.4 to 3.3). Comparing the alternative approaches with the former two-phase experimental layout, the MVD_(F)_ of designs implemented in each scenario was greater than the smallest MVD_(F)_ obtained by model (7) *a*–*c*, except for *Scenario I* (**Tables 5, 6**). Generally, a reduction in MVD_(R)_ of over 0.5 was realized by every alternative two-phase design compared to the previous one (**Tables 5, 6**). Relevant differences in MVD between options generating the design in phase-specific order (1) or across phases (2) were not observed, except for *Scenario I*.

**Table 6:** Two options for evaluating MVD across five scenarios where MVD is obtained by assuming blocks either to be fixed or random in Model (4)^†^ and setting block variances to values of estimated VCs^‡^ to obtain the MVD_(R)_.

Option	Scenario	MVD_(F)_	MVD_(R)_
1	*I*	2.41303	2.08430
	*II*	3.34630	2.11680
2	*III*	3.32754	2.11686
	*IV*	3.33052	2.11687
	*V*	3.37687	2.11794

*^†^ Model (4): IBD in P1, IBD in P2;*

*^‡^The VCs which were listed under the Model (4) in **Table 5** were used as block effects and residual error for random terms to obtain the MVD_*(R)*_.*

#### Two-Phase Designs Containing Different Block Structures in Both Phases

Alternative two-phase designs considering in each phase a different block structure achieved a reduction especially in MVD_(R)_ from about 2.09 to 1.99 in comparison to alternative approaches using the same block structure in both phases, where the minimum MVD_(R)_ was about 2.09. (**Tables 6, 7**). The reduction in MVD_(F)_ was only from 2.41 to 2.35 when in the first phase the only block effect was the “*worker-day*” (*Scenario XIII a–c*). Comparing the options to generate two-phase designs in phase-specific order (1) or across the phases (2) considering different block structures in each phase, the smallest MVD were always found for Option 2 and with the approach using of a single pseudo level for incomplete blocks of P1 and P2 (**Table 7**).

**Table 7:** Two options for evaluating MVD across five scenarios where MVD is obtained by assuming blocks either to be fixed or random and setting block variances to values of estimated VCs^†^ to obtain the MVD_(R)_.

Option	Model^‡^	Scenario	MVD_(F)_	MVD_(R)_
1	5	*VI*	3.36418	2.06195
	6, *a*	*VII - a*	3.27783	2.00898
	6, *b*	*VII - b*	3.25169	1.99296
	6, *c*	*VII - c*	3.29339	2.00670
	7, *a*	*VIII - a*	2.38759	2.01776
	7, *b*	*VIII - b*	2.36051	1.99974
	7, *c*	*VIII - c*	2.36826	2.01205
2	5	*IX*	3.33725	2.06106
	5	*X*	3.39264	2.06423
	6, *a*	*XI - a*	3.24335	2.0075
	6, *b*	*XI - b*	3.21642	1.99175
	6, *c*	*XI - c*	3.26363	2.00558
	6, *a*	*XII - a*	3.32675	2.01238
	6, *b*	*XII - b*	3.29614	1.9959
	6, *c*	*XII - c*	3.33943	2.00878
	7, *a*	*XIII - a*	2.37642	2.01637
	7, *b*	*XIII - b*	2.35226	1.99852
	7, *c*	*XIII - c*	2.36343	2.01112
	7, *a*	*XIV - a*	2.39635	2.01995
	7, *b*	*XIV - b*	2.36992	2.00176
	7, *c*	*XIV - c*	2.37746	2.01321

*^†^Variance components are given in **Table 5**. Those VCs were used as block effects and residual error for random terms to obtain the MVD_*(R)*_, which were listed under the respective models in **Table 5**.*

*^‡^Model (5): IBD in P1 with the additional *post-blocking* factor column, IBD in P2; Model (6) *a*–*c*: IBD with the additional *post-blocking* factor column and “*worker-day*”, where a to c indicate the different blocking strategies to account for the different numbers of position that can be visited by a worker on a day; Model (7) *a*–*c*: considering only the “*worker-day*” block in P1 and an IBD in P2.*

## Discussion

We investigated several options for generating two-phase designs using a model-based design package. These options were explored for the case of an experiment with *P. zonale*, but our key findings are applicable to other crops and two-phase experiment settings, especially with large treatment numbers in breeding. We used the OPTEX package of SAS, but other packages can be used as well.

Our results show that there is great potential for improving the two-phase design in *P. zonale* considering additional blocking factors such as “*worker-day*”, using computer generated designs in both phases rather than conducting the randomization on-site, including equal block sizes in the second phase and extending the generation procedure across phases.

In detail, reductions in MVD were obtained by the use of additional block factors accounting better for environmental variation. For example, a reduction in MVD_(F)_ from 9.42 to 4.41 or in MVD_(R)_ from 2.67 to 2.52 was achieved by considering a column factor and the “*worker-day*” in P1 (**Table 5**). The MVD varied according to the chosen level of the “*worker-day*” factor to define the number of plants a worker may visit per day. For the *b* strategy, the smallest MVD was always obtained independently of the options, indicating that this strategy best represented a day of a worker (**Tables 5–7**).

Further differences in options were identified when different block structures in both phases were considered. In particular, the approach using a single pseudo level for incomplete blocks of P1 and P2, and different block structures in the two phases, realized always the smallest MVD.

### The MVD as the Evaluation Criterion

For the interpretation of the reduction in MVD when comparing the alternative approaches with the two-phase design in 2013/14, the idealized conditions need to be acknowledged. The reduction in the number of incomplete blocks leads to an increased number of direct genotype comparisons within incomplete blocks which also reduces the MVD.

As expected, the MVD_(R)_ was always smaller than the MVD_(F)_ as the estimation of MVD_(R)_ is based not only on the within-block genotype differences (i.e., intra-block information) to obtain adjusted means like for the MVD_(F)_, but also on the information of block sums (i.e., the inter-block information). This stresses the importance of considering the joint inter-block–intra-block analysis (Mead et al., 2012), which can be implemented by taking blocks as random, during the design evaluation (Möhring et al., 2015). Note that, when instead of the VC of the former experiment, very large values are used for the variance of block effects, while leaving the value of residual error variance unchanged during the dummy analysis, i.e., there is no inter-block information, then values of MVD_(R)_ and MVD_(F)_ coincide. Further, the MVD_(R)_ varies depending on the values of VCs for block effects considered in dummy analyses, but the ranks of scenarios remained unchanged in the cases we investigated (**Supplementary Presentation 4**).

### The Need for Randomization

Randomization is conducted to avoid systematic effects and other biases in single-phase experiments (Piepho et al., 2013). In two phase experiments, these problems exist in both phases and therefore randomization should be carried out in both phases. In *Scenario I*, we omitted randomization in the second phase and increased thereby the efficiency of analysis, because only one incomplete block adjustment was needed to estimate the genotypes effects across the two phases [compare Model (2) and Model (4)]. That is why *Scenario I* showed the smallest MVD compared to the other designs assuming in each phase the same block structure. In conclusion, it is actually advantageous to transmit the experimental layout from one phase to the other whenever possible.

### Best Options for Generating Two-Phase Designs and Application to Other Breeding Trials

Two-phase designs should be generated across phases (Option 2) rather than in phase-wise order (Option 1) to guarantee the smallest MVD, which was most frequently obtained for Option 2. The only exception was *Scenario I*. A reason for the better performance of Option 2 is that the block structure in both phases is taken into account simultaneously when sets of genotypes are allocated to them. Thus, treatment concurrences occur equally often or only once across phases in an optimized two-phase design (**Supplementary Presentation 4**), which is known to be optimal in single phase experiment (John and Williams, 1995). Further, we demonstrated in the Scenarios *XIII* to *XIV* under Option 2 that our proposed approaches for generating two-phase designs across phases can be adjusted to any block size in each phase if necessary, making our approaches relevant to a broad range of applications. For the generation of a two-phase design with eight replicates (*Scenario III*) a similar MVD_(R)_ or even a smaller MVD_(F)_ was found than for the approach using a phase specific dummy variable (*Scenario IV*). But *Scenario III* was not further considered, as this approach restricts block structures to be the same in both phases. However, *Scenario III* represents an option for estimating of VCs for design effects in each phase is of interest when the structure in both phases is the same.

Examples for the application of our approaches in other ornamental species are the evaluation of rooting in P1 and other phenotypic traits in P2 in *Osteospermum* or the evaluation of germination rate and flowering time in the first and second phase in *Dianthus* ssp. (Selecta one). In the former example, in each phase the same block structure was considered, whereas in the latter example, in each phase a different block was assumed.

### Consideration of Worker-Days as a Block Factor

The greatest reduction in MVD was obtained when we accounted for the worker-induced variation by blocks in *post hoc* analysis of the previous experiment by models (6), *a–c* to (7), *a–c* (**Table 5**), although the reduction of error variance was relatively small (**Table 4**). This shows that workers are a source of variation and reaffirms the recommendation that known sources of variation should be captured by blocking and considered before the experiment is conducted, as precision of genotype comparisons will be increased (Mead et al., 2012).

### Idealized Conditions in Practice

The notable reduction in MVD_(F)_ and MVD_(R)_ realized by the alternative approaches justifies the implementation of idealized conditions in the *P. zonale* breeding program, especially the use of a pre-defined layout in the second phase. Under these idealized conditions, the breeder needs to randomly select six out of the total of harvested stem cuttings per pair of stock plants and genotypes in P1, which shall be rooted in P2. The procedure of packaging genotypes remains essentially the same as in the previous experiment, where the harvested stem cuttings of each genotype and replicate are packed in small bags such that each bag contained the six randomly selected stem cuttings from the EU1 in the first phase. However, the bags are now ordered according to the planting positions in P2 and then packed into cartons, where genotypes are grouped by replicate. In P2, an efficient workflow is ensured and hence, plant quality is maintained as workers plant genotypes onto trays according the planting number.

## Conclusion

With respect to the considered options, our results show that two-phase designs should be generated across phases (Option 2) rather than in phase-wise order (Option 1) to guarantee the smallest MVD, which was obtained for Option 2 with different block structures in both phases and the approach using a single pseudo level for incomplete blocks in P1 and P2. Increase in efficiency can be expected when the experimental layout is transmitted from P1 to P2.

With our pragmatic approaches, we could improve the present two-phase design in *P. zonale* breeding, which yields a reduction in the MVD obtained by intra-block analysis from 9.42 to about 2.35 or obtained by combined inter-block–intra-block analysis from 2.67 to approximately 1.99 by using computer generated designs in both phases rather than conducting the randomization on-site, additional block factors in P1, and extending the generation procedure across phases. This significant reduction in MVD justifies the consideration of idealized conditions in *P. zonale* breeding and indicates that the on-site randomization approach is sub-optimal. The proposed alternative approaches can be transferred to other studies that involve two-phase experimental set-ups and they can be implemented in any model-based design package with facilities to freely formulate linear models for treatment and block structures.

## Author Contributions

HM and H-PP developed alternative methods generating the two-phase experimental designs. HM conducted *post hoc* analysis of the experiment 2013/14, the dummy analyses and prepared the manuscript. H-PP and RB revised the manuscript. All authors discussed the results, commented on the manuscript and approved the final manuscript.

## Conflict of Interest Statement

The authors declare that the research was conducted in the absence of any commercial or financial relationships that could be construed as a potential conflict of interest.

## Supplementary Material

PRESENTATION 1 Coder for generating two-phase designs.

PRESENTATION 2 Figures of approaches implemented in Scenarios I to V.

PRESENTATION 3 Codes for dummy analyses.

PRESENTATION 4 Concurrence of two-phase designs.

## Abbreviations

EU1: the experimental unit in P1
EU2: the experimental unit in P2
IBD: incomplete block design
MVD: the mean variance of a pair-wise treatment difference
MVD_(F)_: the MVD obtained by the intra-block analysis
MVD_(R)_: the MVD obtained by the joint inter-block-intra-block analysis
P1: phase one
P2: phase two
VC: variance components
