# Supplementary material for: Identifying Effective Design Approaches to Allocate Genotypes in Two-Phase Designs: A Case Study in Pelargonium zonale
Source: Front Plant Sci. 2018 Jan 5;8:2194. doi: 10.3389/fpls.2017.02194 (PMC5760546; doi:10.3389/fpls.2017.02194)
Supplement: PRESENTATION 1 — Coder for generating two-phase designs. [file Presentation_1.PDF]

Codes to generate two-phase experimental designs

Code 1 is for generating a resolvable IBD with *v* = 504, *r* = 4, *b* = 168 and *k* = 3 using the procedure OPTEX with a detailed description of each step. Based on Code 1, modifications were made for the implementations of all other scenarios.
For the reproducibility of each scenario the specific randomization seed value is given.

1 The same block structure in both phases

1.1 Option 1 – Design generation for each phase separately

Code 1 – Scenario I

An OPTEX code used to generate a resolvable IBD with *v* = 504, *r* = 4, *b* = 168 and *k* = 3 may be divided into three steps:

1) Generating a data file for defining the treatment labels.

```
DATA TRT;
DO GENO=1 TO 504;
OUTPUT;
END;
RUN;
```

2) Generating a layout file for defining the incomplete block and plot labels for the physical experimental plan.

```
DATA LAYOUT;
DO REP=1 TO 4;
DO IB=1 TO 84;
DO PLOT=1 TO 6;
OUTPUT;
END;
END;
END;
RUN;
```

3) Assigning both, the treatment and the layout files to the OPTEX code to generate the design for the specified design parameters.

```
PROC OPTEX DATA=TRT SEED=345;
CLASS GENO;
```

```
MODEL GENO;
BLOCKS DESIGN=LAYOUT NITER=10 KEEP=10;
CLASS REP IB PLOT;
MODEL REP, IB(REP)/ PRIOR=0, 2016;
OUTPUT OUT=SCENARIO_1;
RUN;
```

Assuming a positive seed to the random number generator (SEED= option) allows an exact reproduction of the design with our code. The treatment model and the block model are stated separately. Design optimization focuses on the treatment model, but takes into account the block structure and assigns treatments to blocks in a way that maximizes efficiency (Piepho, 2015). In this case, the optimal solution is to assign each treatment to each block exactly once. The file with the treatment labels is provided via the DATA= option of the procedure OPTEX, followed by the CLASS statement and the treatment MODEL. The layout file representing the physical experimental plan is provided via the BLOCKS statement, where a second CLASS statement specifies the design parameters of the BLOCK model. In the BLOCKS statement one can specify the number of iterations by the NITER= option to find the optimal design, numbers of iterations to be kept by is specified by the KEEP= option. Further, in the block model PRIOR values are assigned for the variance of block effects as proposed by Pereira and Tobias (2015). The approach behind the PRIOR option is a Bayesian concept and described in DuMouchel and Jones (1994). The approach allows to assign weights on both to the effects in the treatment model as well as to effects in the block model. Here we used the total number of experimental units of the two-phase experiments. As the experimental units of P1 were totally confounded with the experimental units of P2, we found 2016 experimental units across the two phases. If necessary, we increased the prior value till we found a resolvable design.
The design file generated by this code is displayed by the OUTPUT statement.
For more details see the procedure OPTEX manual (https://support.sas.com/documentation/onlinedoc/qc/132/optex.pdf).

Code 2 – Scenario II

To generate two different experimental layouts, only the randomization SEED value was changed in the OPTEX code.

```
/*Design Phase 1*/
PROC OPTEX DATA=TRT SEED=236;

/*Design Phase 2*/
PROC OPTEX DATA=TRT SEED=987;
```

1.2 Option 2 – Design generation across the two phases

Code 3 – Scenario III – Increasing the replicate number

The $r = 4$ replicates were increased to $r = 8$ replicates, whereas all other design parameters remain unchanged. Then, the 8 replicates were split among the two phases.

```
PROC OPTEX DATA=TRT SEED=499;


DATA LAYOUT;
DO REP=1 TO 8;
DO IB=1 TO 84;
DO PLT=1 TO 6;
OUTPUT;
END;
END;
END;
RUN;
```

Code 4 – Scenario IV – Separation of block structures using phase-specific dummy coding

Two layout files were defined, one for each phase one. Then the data files of the two layouts were merged, and the incomplete blocks of P1 are coded as 0 for P2 and vice versa. Further, the incomplete blocks of both phases need to be assigned to second CLASS and MODEL statement of the OPTEX code.

```
DATA PHASE1;
DO PHASE= 1 TO 1;
DO REP=1 TO 4;
DO IB1=1 TO 84;
DO PLT=1 TO 6;
OUTPUT;
END;
END;
END;
END;
RUN;

DATA PHASE2;
DO PHASE= 2 TO 2;
DO REP=1 TO 4;
```

```
DO IB2=1 TO 84;
DO PLT=1 TO 6;
OUTPUT;
END;
END;
END;
END;
RUN;

DATA TP;
SET PHASE1 PHASE2;
IF PHASE=1 THEN IB2=0;
IF PHASE=2 THEN IB1=0;
RUN;
```

An excerpt of the resulting layout file "TP"

**PHASE**	REP	IB1	IB2	PLOT
**1**	1	1	**0**	1
**1**	1	1	**0**	2
**1**	1	1	**0**	3
**1**	1	2	**0**	1
**1**	1	2	**0**	2
**1**	1	2	**0**	3
**2**	1	**0**	1	1
**2**	1	**0**	1	2
**2**	1	**0**	1	3
**2**	1	**0**	2	1
**2**	1	**0**	2	2
**2**	1	**0**	2	3

```
PROC OPTEX DATA=TRT SEED=493;
CLASS GENO;
MODEL GENO;
BLOCKS DESIGN=TP NITER=10 KEEP=10;
CLASS PHASE REP IB1 IB2;
MODEL PHASE REP, IB1(REP), IB2(REP) /PRIOR=0, 2016 ;
OUTPUT OUT=SCENARIO_4;
RUN;
```

Code 5 – Scenario V – Two step approach: i) The allocation incomplete blocks of P2 to incomplete blocks of P1 in replicate-wise order and ii) the allocation of genotypes to the block structure of both phases

In the first step, the block structure of P2 is defined in the first treatment file, and the block structure of P1 is defined in the layout file. Both files are then assigned to the OPTEX code to

allocate the incomplete blocks of P2 to the incomplete blocks of P1. This is done for each replicate separately. In the following, an example for replicate 1 is given. For each replicate a new randomization SEED value is used. The design files of each replicate are then concatenated to obtain the layout file including all replicates.

In the second step, the genotype labels are defined in a second layout file and then assigned with the design file, comprising all replicates, to a second OPTEX code to allocate genotypes to the block structure of P1 and P2.

To facilitate use of the proposed method, a macro is given below – Macro V. This macro allows performing Step I in such a way that not for every replicate, an OPTEX code needs to be written. However, the treatment files and layout file need to be defined before running the macro.

```
/*STEPI*/
/*First treatment file: Definition of the block structure in P2*/
DATA TRT;
DO IB2=1 TO 84;
OUTPUT;
END;
RUN;

/*Layout file: Definition of the block structure in P1*/
DATA PHASE1;
DO IB1=1 TO 84;
DO PLT=1 TO 6;
OUTPUT;
END;
END;
RUN;

/*REPLICATE 1*/
PROC OPTEX DATA=TRT SEED=3149;
CLASS IB2;
MODEL IB2;
BLOCKS DESIGN=PHASE1;
CLASS IB1;
MODEL IB1;
OUTPUT OUT= REP1;
RUN;


/*REPLICATE 2*/
PROC OPTEX DATA=TRT SEED=8745;

/*REPLICATE 3*/
PROC OPTEX DATA=TRT SEED=5841;

/*REP 4*/
```

PROC OPTEX DATA=TRT SEED=1846;

/*STEPII*/

/*Second treatment file: Definition of genotype labels***/**

DATA TRT2;

```
DO GENO= 1 TO 504;
OUTPUT;
END;
RUN;
```

PROC OPTEX DATA=TRT2 SEED=6198;

```
CLASS GENO;
MODEL GENO;
BLOCKS DESIGN=LAYOUT NITER=10 KEEP=10;
CLASS IB1 IB2 REP;
```

MODEL REP, IB1(REP) IB2(REP)/PRIOR=0,1E6;

```
OUTPUT OUT=SCENARIO_5;
RUN;
```

Scenario V MACRO

/*First treatment file: Definition of the block structure in P2*/

DATA TRT;

```
DO IB2=1 TO 84;
OUTPUT;
END;
RUN;
```

/*Layout file: Definition of the block structure in P1*/

```
DATA PHASE1;
DO IB1=1 TO 84;
DO PLT=1 TO 6;
OUTPUT;
END;
END;
RUN;
```

/*Second treatment file: Definition of genotype labels***/**

DATA TRT2;

```
DO GENO= 1 TO 504;
OUTPUT;
END;
RUN;
%MACRO REIBD (SEED, OUT1);
```

PROC OPTEX DATA=TRT SEED=&SEED;

```
CLASS IB2;
MODEL IB2;
```

```
BLOCKS DESIGN=PHASE1;
CLASS IB1;
MODEL IB1;
OUTPUT OUT=&OUT1;
RUN;
%MEND REIBD;

/*Specifying the randomization seed value for each replicate*/
%REIBD(3149, REP1)
%REIBD(8745, REP2)
%REIBD(5841, REP3)
%REIBD(1846, REP4)

%MACRO RENR (REP, OUT1, OUT2);
DATA &OUT2; SET &OUT1; REP=&REP; RUN;
%MEND RENR;

%RENR(1, REP1, REP1)
%RENR(2, REP2, REP2)
%RENR(3, REP3, REP3)
%RENR(4, REP4, REP4)

DATA LAYOUT; SET REP1 REP2 REP3 REP4; RUN;

PROC OPTEX DATA=TRT2 SEED=6198;
CLASS GENO;
MODEL GENO;
BLOCKS DESIGN=LAYOUT NITER=10 KEEP=10;
CLASS IB1 IB2 REP;
MODEL REP, IB1(REP) IB2(REP)/PRIOR=0,1E6;
OUTPUT OUT=SCENARIO_5;
RUN;
```

2 Different block structures in both phases

2.1 Option 1 – Design generation for each phase separately

Code 6 – Scenario VI – Row-column design in P1, resolvable IBD in P2

The layout file was changed to 84 rows and 6 columns within each replicate. To the OPTEX code in the second CLASS and MODEL statement the incomplete block of P1 were replaced by the rows and columns.
The resolvable IBD generated by code 1 was used for the second phase.

```
DATA LAYOUT;
DO REP=1 TO 4;
DO ROW=1 TO 84;
DO COL=1 TO 6;
OUTPUT;
END;
END;
END;
RUN;

PROC OPTEX DATA=TRT SEED=345;
CLASS GENO;
MODEL GENO;
BLOCKS DESIGN=LAYOUT NITER=10 KEEP=10;
CLASS REP IB PLT ROW COL;
MODEL REP, ROW(REP) COL(REP) IB2(REP) /PRIOR=0, 2016 ;
OUTPUT OUT=SCENARIO_6;
RUN;
```

Code 7 – Scenario VII – Considering the additional block factor "worker-day" in P1, retaining the resolvable IBD in P2

To consider the "*worker-day*" during the design generation, the additional block factor "*worker-day*" is defined using the CEIL function on functions of the ROW and COL factors, to round up to the nearest integer. In D1, the number of rows and D2, the number of columns, are considered, which are grouped together by the "*worker-day*" according to the three blocking strategies *a*, *b* and *c* (Table 3). The additional block factor is added to the second CLASS and MODEL statements of the OPTEX code. The generated design of *Scenario I* is used in P2 (Code 1).

PROC OPTEX DATA=TRTLAB SEED=345;

Scenario VII – a

DATA LAYOUT;
DO REP=1 TO 4;
DO ROW=1 TO 84;
D1=CEIL(ROW/21);
DO COL=1 TO 6;
D2=CEIL(COL/3);
WORK=(D1-1)*2+D2;
OUTPUT;
END;
END;
END;
END;
RUN;

Scenario VII – b

DATA LAYOUT;
DO REP=1 TO 4;
DO ROW=1 TO 84;
D1=CEIL(ROW/28);
DO COL=1 TO 6;
D2=CEIL(COL/3);
WORK=(D1-1)*2+D2;
OUTPUT;
END;
END;
END;
END;
RUN;

Scenario VII – c

DATA LAYOUT;
DO REP=1 TO 4;
DO ROW=1 TO 84;
D1=CEIL(ROW/42);
DO COL=1 TO 6;
D2=CEIL(COL/3);
WORK=(D1-1)*2+D2;
OUTPUT;
END;
END;
END;
END;
RUN;

PROC OPTEX DATA=TRT SEED=345;
CLASS GENO;

```
MODEL GENO;
BLOCKS DESIGN=LAYOUT NITER=10 KEEP=10;
CLASS REP ROW COL WORK;
MODEL REP, WORK(REP) ROW(REP) COL(REP)/ PRIOR=0,2016;
OUTPUT OUT=SCENARIO_7;
RUN;
```

Code 8 – Scenario VIII – Considering only the block factor "worker-day" in P1 and retaining the resolvable IBD in P2

By considering only the "*worker-day*" and incomplete block effects in P1 and P2, the proportion of variance explained by those effects were maximized and hence we considered in each phase only one block factor. To consider only the block factor "*worker-day*", the definition of that block factor was done using the rows and columns of the row-column design (see for comparison Code 7). However, when the design is generated, the rows and columns are neglected resulting in the following block model in the OPTEX procedure. The resolvable IBD generated by the use of Code 1 was considered in P2.

```
PROC OPTEX DATA=TRTLAB SEED=345;

/*BLOCK STRUCTURE P1*/
/*EXAMPLE FOR BLOCK STRATEGY A*/
DATA LAYOUT;
DO REP=1 TO 4;
DO ROW=1 TO 84;
D1=CEIL(ROW/21);
DO COL=1 TO 6;
D2=CEIL(COL/3);
WORK=(D1-1)*2+D2;
OUTPUT;
END;
END;
END;
END;
RUN;

PROC OPTEX DATA=TRT SEED=345;
CLASS GENO;
MODEL GENO;
BLOCKS DESIGN=LAYOUT NITER=10 KEEP=10;
CLASS REP WORK;
MODEL REP, WORK(REP) / PRIOR=0,2016;
OUTPUT OUT=SCENARIO_8;
RUN;
```

2.2 Option 2 – Design generation across phases

Code 9 – Scenario IX – Row-column design in P1 and retaining the resolvable IBD in P2 by the use of the separation of block structures using a phase-specific dummy coding

The layout file, in which the block structure of P1 is defined, is changed to generate a row-column design with 84 rows and 6 columns. Now, the rows and columns are set to zero for incomplete blocks of P2. The incomplete block of P1 is replaced by the rows and columns in the second CLASS and MODEL statement.

```
DATA PHASE1;
DO PHASE=1 TO 1;
DO REP=1 TO 4;
DO ROW=1 TO 84;
DO COL=1 TO 6;
OUTPUT;
END;
END;
END;
END;
RUN;

PROC OPTEX DATA=TRT SEED=345;
CLASS GENO;
MODEL GENO;
BLOCKS DESIGN=LAYOUT NITER=10 KEEP=10;
CLASS PHASE REP ROW COL IB2;
MODEL PHASE REP, ROW(REP) COL(REP) IB2(REP)/ PRIOR=0,2016;
OUTPUT OUT=SCENARIO_9;
RUN;
```

Code 10 – Scenario X - Row-column design in P1 and retaining the resolvable IBD in P2 generated using the two-step approach by i*)* The allocation incomplete blocks in P2 to rows and columns of P1 in replicate-wise order and ii*)* the allocation of genotypes to the block structure of both phases

Only the layout file is modified to generate a row-column design with 84 rows and 6 columns. The treatment files remain unchanged. The incomplete blocks of P2 are now allocated to rows and columns in P1, that is why the incomplete blocks of P1 were replaced by rows and columns in the second CLASS and MODEL statement of the first OPTEX code. In the second OPTEX code, the incomplete blocks of P1 were replaced by rows and columns to allocate the genotypes to rows and columns of P1 as well as to incomplete blocks of P2.

```
/*BLOCK STRUCTURE P1*/
DATA PHASE1;
REP=1;
DO ROW=1 TO 84;
DO COL=1 TO 6;
OUTPUT;
END;
END;
RUN;
```

Scenario X Macro

```
%MACRO REIBD (SEED, OUT1);
PROC OPTEX DATA=TRT SEED=&SEED;
CLASS IB2;
MODEL IB2;
BLOCKS DESIGN=PHASE1;
CLASS ROW COL;
MODEL ROW COL;
OUTPUT OUT=&OUT1;
RUN;
%MEND REIBD;

/*SPECIFYING THE RANDOMIZATION SEED VALUE FOR EACH
REPLICATE*/
%REIBD(3149, REP1)
%REIBD(8745, REP2)
%REIBD(5841, REP3)
%REIBD(1846, REP4)

%MACRO RENR (REP, OUT1, OUT2);
DATA &OUT2; SET &OUT1; REP=&REP; RUN;
%MEND RENR;

%RENR(1, REP1, REP1)
%RENR(2, REP2, REP2)
%RENR(3, REP3, REP3)
%RENR(4, REP4, REP4)
DATA LAYOUT; SET REP1 REP2 REP3 REP4; RUN;

PROC OPTEX DATA=TRT2 SEED=6198;
CLASS GENO;
MODEL GENO;
BLOCKS DESIGN=LAYOUT NITER=10 KEEP=10;
```

```
CLASS ROW COL IB2 REP;
MODEL REP, ROW(REP) COL(REP) IB2(REP)/PRIOR=0,1E6;
OUTPUT OUT=SCENARIO_10;
RUN;
```

Code 11 – Scenario XI – Row-column design with the additional block factor "worker-day" in P1 and retaining the resolvable IBD in P2 using separation of block structures by the use of phase-specific dummy coding

The layout file, in which the block structure of the P1 is defined, is changed to generate a row-column design with 84 rows and 6 columns and considering the additional block factor "*worker-day*" in the same manner as in *Scenario VII*. The additional block factor is also set to zero and added to the second CLASS and MODEL statement of the OPTEX code. The remaining code is unchanged.

```
/*BLOCK STRUCTURE P1*/
/*EXAMPLE FOR BLOCK STRATEGY A*/
DATA PHASE1;
DO REP=1 TO 4;
DO ROW=1 TO 84;
D1=CEIL(ROW/21);
DO COL=1 TO 6;
D2=CEIL(COL/3);
WORK=(D1-1)*2+D2;
OUTPUT;
END;
END;
END;
END;
RUN;

PROC OPTEX DATA=TRT SEED=345;
CLASS GENO;
MODEL GENO;
BLOCKS DESIGN=LAYOUT NITER=10 KEEP=10;
CLASS PHASE REP WORK ROW COL IB2;
MODEL PHASE REP, WORK(REP) ROW(REP) COL(REP) IB2(REP)/
PRIOR=0,2016;
OUTPUT OUT=SCENARIO_11;
RUN;
```

Code 12 – Scenario XII - Row-column design with the additional block factor "worker-day" in P1 and retaining the resolvable IBD in P2 by i*)* The allocation of incomplete blocks of P2 to rows, columns and "worker-day" of P1 in replicate-wise order and ii*)* the allocation of genotypes to the block structure of both phases

The layout file is modified to generate a row-column design with 84 rows and 6 columns, which were grouped together by the additional block factor "*worker-day*". The treatment files remain unchanged. The incomplete blocks of P2 are allocated not only to rows and columns in P1 but also to the additional block factor "*worker-day*". Therefore, the block factor "worker-day" was added to the second CLASS and MODEL statement of the first and second OPTEX code.

```
/*BLOCK STRUCTURE P1*/
/*EXAMPLE FOR BLOCK STRATEGY A*/
DATA PHASE1;
DO REP=1 TO 4;
DO ROW=1 TO 84;
D1=CEIL(ROW/21);
DO COL=1 TO 6;
D2=CEIL(COL/3);
WORK=(D1-1)*2+D2;
OUTPUT;
END;
END;
END;
END;
RUN;
```

Scenario XII Macro

```
%MACRO REIBD (SEED, OUT1);
PROC OPTEX DATA=TRT SEED=&SEED;
CLASS IB2;
MODEL IB2;
BLOCKS DESIGN=PHASE1;
CLASS WORK ROW COL;
MODEL WORK ROW COL;
OUTPUT OUT=&OUT1;
RUN;
%MEND REIBD;

/*SPECIFYING THE RANDOMIZATION SEED VALUE FOR EACH
REPLICATE*/
%REIBD(3149, REP1)
%REIBD(8745, REP2)
%REIBD(5841, REP3)
%REIBD(1846, REP4)

%MACRO RENR (REP, OUT1, OUT2);
DATA &OUT2; SET &OUT1; REP=&REP; RUN;
%MEND RENR;
```

```
%RENR(1, REP1, REP1)
%RENR(2, REP2, REP2)
%RENR(3, REP3, REP3)
%RENR(4, REP4, REP4)

DATA LAYOUT; SET REP1 REP2 REP3 REP4; RUN;
PROC OPTEX DATA=TRT2 SEED=6198;
CLASS GENO;
MODEL GENO;
BLOCKS DESIGN=LAYOUT NITER=10 KEEP=10;
CLASS ROW COL IB2 REP WORK;
MODEL REP, WORK(REP) ROW(REP) COL(REP) IB2(REP)/PRIOR=0,1e6;
OUTPUT OUT=SCENARIO_12;
RUN;
```

Code 13 – Scenario XIII – Considering only the block factor "worker-day" in P1 and retaining the resolvable IBD in P2 using separation of block structures across phases by the use of phase-specific dummy coding

By considering only the "*worker-day*" and incomplete block effects in P1 and P2, the proportion of variance explained by those effects was maximized and hence we considered in each phase only one of each block factor. To consider the block factor "*worker-day*", the factor WORK was assigned to the BLOCK statement (see for comparison Code 7).

```
/*BLOCK STRUCTURE P1*/
/*EXAMPLE FOR BLOCK STRATEGY A*/
DATA PHASE1;
DO REP=1 TO 4;
DO ROW=1 TO 84;
D1=CEIL(ROW/21);
DO COL=1 TO 6;
D2=CEIL(COL/3);
WORK=(D1-1)*2+D2;
OUTPUT;
END;
END;
END;
END;
RUN;

PROC OPTEX DATA=TRT SEED=345;
CLASS GENO;
MODEL GENO;
BLOCKS DESIGN=LAYOUT NITER=10 KEEP=10;
CLASS PHASE REP WORK IB2;
MODEL PHASE REP, WORK(REP) IB2(REP)/ PRIOR=0,2016;
OUTPUT OUT=SCENARIO_13;
RUN;
```

Code 14 – Scenario XIV – Considering only the block factor "worker-day" in P1 and retaining the resolvable IBD in P2 by i) The allocation of incomplete blocks of P2 to rows, columns and "worker-day" of P1 in replicate-wise order and ii) the allocation of genotypes to the block structure of both phases

By considering only the "*worker-day*" and incomplete block effects in P1 and P2, the proportion of variance explained by those effects was maximized and hence we considered in each phase only one of each block factor in each phase. The block factor "*worker-day*, only WORK was assigned to the BLOCK statement (see for comparison Code 7).

```
/*BLOCK STRUCTURE P1*/
/*EXAMPLE FOR BLOCK STRATEGY A*/
DATA PHASE1;
DO REP=1 TO 4;
DO ROW=1 TO 84;
D1=CEIL(ROW/21);
DO COL=1 TO 6;
D2=CEIL(COL/3);
WORK=(D1-1)*2+D2;
OUTPUT;
END;
END;
END;
END;
RUN;
```

Scenario XI Macro

```
%MACRO REIBD (SEED, OUT1);
PROC OPTEX DATA=TRT SEED=&SEED;
CLASS IB2;
MODEL IB2;
BLOCKS DESIGN=PHASE1;
CLASS WORK;
MODEL WORK;
OUTPUT OUT=&OUT1;
RUN;
%MEND REIBD;
```

/*SPECIFYING THE RANDOMIZATION SEED VALUE FOR EACH REPLICATE*/

```
%REIBD(3149, REP1)
%REIBD(8745, REP2)
%REIBD(5841, REP3)
%REIBD(1846, REP4)

%MACRO RENR (REP, OUT1, OUT2);
DATA &OUT2; SET &OUT1; REP=&REP; RUN;
%MEND RENR;

%RENR(1, REP1, REP1)
%RENR(2, REP2, REP2)
%RENR(3, REP3, REP3)
%RENR(4, REP4, REP4)

DATA LAYOUT; SET REP1 REP2 REP3 REP4; RUN;
```

PROC OPTEX DATA=TRT2 SEED=6198;

```
CLASS GENO;
MODEL GENO;
BLOCKS DESIGN=LAYOUT NITER=10 KEEP=10;
```

CLASS IB2 REP WORK;

MODEL REP, WORK(REP) IB2(REP)/PRIOR=0,1E6;

```
OUTPUT OUT=SCENARIO_14;
RUN;
```
