# Supplementary material for: Identifying Effective Design Approaches to Allocate Genotypes in Two-Phase Designs: A Case Study in Pelargonium zonale
Source: Front Plant Sci. 2018 Jan 5;8:2194. doi: 10.3389/fpls.2017.02194 (PMC5760546; doi:10.3389/fpls.2017.02194)
Supplement: PRESENTATION 2 — Figures of approaches implemented in Scenarios I to V. [file Presentation_2.PDF]

Dummy analysis

Dummy response

The response variable was set to an arbitrary value. In this case to unity.

```
DATA <layout file> ; SET <layout file> ;
DUMMY_RESPONSE=1;
RUN;
```

The intra-block analysis

For the intra-block analysis of two-phase designs containing the same block structure in both phases, in the MIXED procedure the data file containing the experimental layout is invoked by the DATA statement. In the CLASS statement all categorical factors are listed. For the intra-block analysis, all block and treatment factors are assigned in the MODEL statement, because all of them are assumed to be fixed effects (bold-faced in the code). The residual error variance is fixed to the value obtained from the previous experiment 2013/14 (bold-faced in the code) in the PARMS statement and HOLD option. In the LSMEANS statement the factor for which the pair-wise differences should be obtained, is written, and differences obtained by the PDIFF option. By the output delivery system ODS the results of the pair-wise genotype comparison are rooted to a SAS file, containing, *inter alia*, the standard error of a difference per genotype comparison. To get the mean variance of the difference (MVD), the standard errors of pair-wise comparisons are squared and then averaged.

```
ODS OUTPUT DIFFS=DIFFS COVPARMS=CP;
PROC MIXED DATA=<layout file>;
CLASS GENO IB1 REP IB2;
MODEL DUMMY_RESPONSE = GENO REP REP*IB1 REP*IB2;
PARMS (3.7806)/HOLD=1;
LSMEANS GENO/PDIFF;
RUN;

DATA DIFFS;
SET DIFFS;
VD =STDERR**2;
RUN;

PROC MEANS DATA=DIFFS MEAN;
VAR VD;
OUTPUT OUT=MEAN_VD MEAN=MEAN_VD;
RUN;

PROC PRINT DATA=MEAN_VD;
RUN;
```

The joint inter-block-intra-block analysis

For the joint inter-block-intra-block analysis, the MIXED procedure is used. By the DATA option the data file containing the experimental layout is invoked, which shall be evaluated. In the CLASS statement categorical factors are listed. The joint inter-block-intra-block analysis is

conducted by taking all block effects as random, which is done by assigning all block effects to the RANDOM statement. The values of block effects are fixed at the VC estimated from the former experiment by the PARMS statement and HOLD option (bold-faced in the code). Pay attention to the order of the random effects! The values in the PARMS statement need to have the order as the block factors are listed in the RANDOM statement. Only the treatment factor is listed in the MODEL statement, for which the pair-wise comparison is of interest and obtained by the LSMEANS statement and PDIFF option. A summary of the pair-wise genotype comparison is obtained by the output delivery system ODS, which provides all standard errors of a pair-wise genotype comparison. To get the MVD, the standard errors of pair-wise genotype comparisons are squared and then averaged.

```
/*Scenario I to V*/
ODS OUTPUT DIFFS=DIFFS COVPARMS=CP;
PROC MIXED DATA=<layout file>;
CLASS GENO IB1 REP IB2;
MODEL DUMMY_RESPONSE = GENO;
RANDOM REP REP*IB1 REP*IB2;
PARMS (2.6569)(0.1303)(0.5066)(3.7806)/HOLD=1,2,3,4;
LSMEANS GENO/PDIFF;
RUN;

DATA DIFFS;
SET DIFFS;
VD =STDERR**2;
RUN;

PROC MEANS DATA=DIFFS MEAN;
VAR VD;
OUTPUT OUT=MEAN_VD MEAN=MEAN_VD;
RUN;

PROC PRINT DATA=MEAN_VD;
RUN;
```

Different VC in PARMS statement for two-phase designs containing different block structures in each phase

For scenarios, in which two-phase designs considered containing a different block structure in both phases, the values of VC of blocks assigned in the PARMS statement need to be adapted.

```
/*Scenario VI, IX, X*/
/*in first phase row-column, in the second phase IBD*/
RANDOM REP;
RANDOM REP*ROW;
RANDOM REP*COL;
RANDOM REP*IB2;
PARMS (2.6378)(0.0505)(0.2043)(0.4378)(3.7315)/HOLD=1,2,3,4,5;

/*Scenario VII, XI,, XII each a to c*/
/*In first phase row-column considering “worker-day”, in the second phase IBD*/
/*Block strategy a*/
```

```
RANDOM REP*WORK;
RANDOM REP*ROW;
RANDOM REP*COL;
RANDOM REP*IB2;
PARMS (2.6406)(0.2569)(0.0443)(0.0823)(0.4518)(3.5895)/HOLD=1,2,3,4,5,6;

/*Block strategy b*/
RANDOM REP*WORK;
RANDOM REP*ROW;
RANDOM REP*COL;
RANDOM REP*IB2;
PARMS (2.5939)(0.3204)(0.0418)(0.0568)(0.4516)(3.5767)/HOLD=1,2,3,4,5,6;

/*Block strategy c*/
RANDOM REP;
RANDOM REP*WORK;
RANDOM REP*ROW;
RANDOM REP*COL;
RANDOM REP*IB2;
PARMS (2.5796)(0.3097)(0.0172)(0.0497)(0.4616)(3.6381)/HOLD=1,2,3,4,5,6;

/*Scenario VIII, XIII,, XIV each a to c*/
/*In first phase only “worker-day”, in the second phase IBD*/
/*Block strategy a*/
RANDOM REP;
RANDOM REP*WORK;
RANDOM REP*IB2;
PARMS (2.6192)(0.3131)(0.4892)(3.6524)/HOLD=1,2,3,4;

/*Block strategy b*/
RANDOM REP;
RANDOM REP*WORK;
RANDOM REP*IB2;
PARMS (2.5743)(0.3542)(0.4780)(3.6360)/HOLD=1,2,3,4;

/*Block strategy c*/
RANDOM REP;
RANDOM REP*WORK;
RANDOM REP*IB2;
PARMS (2.5621)(0.3352)(0.4854)(3.6729)/HOLD=1,2,3,4;
```
