# Supplementary material for: Identifying Effective Design Approaches to Allocate Genotypes in Two-Phase Designs: A Case Study in Pelargonium zonale
Source: Front Plant Sci. 2018 Jan 5;8:2194. doi: 10.3389/fpls.2017.02194 (PMC5760546; doi:10.3389/fpls.2017.02194)
Supplement: PRESENTATION 3 — Codes for dummy analyses. [file Presentation_3.PDF]

1 Concurrence in two-phase designs

The concurrence matrix $\boldsymbol{M}$ was used either to count the treatment concurrences in blocks across phases of designs implemented in scenarios, where each phase involved one block factor (*Scenario I* to *V*) or phase one comprised two block factors (rows and columns) and phase two one block factor (*Scenarios VI*, *VIII* or *X*).

In cases in which the first phase involved two block factors, *i.e.* rows and columns, first, the treatment concurrences were counted across rows and incomplete blocks and second, across columns and incomplete blocks.

The concurrence matrix, $\boldsymbol{M}$, was defined as,

$$\boldsymbol{M} = \sum_{i=1}^{p}\sum_{j=1}^{r} \boldsymbol{N_{ij}}\boldsymbol{N_{ij}^{T}}$$

where $\boldsymbol{N_{ij}}$ was an incidence matrix of dimension $n \times m$ for the i-th phase (i = 1, p = 2) and j-th replicate (j = 1,…, r = 4), each with n rows for the n treatments and m columns for the m incomplete blocks within a replicate and $\boldsymbol{N_{ij}^{T}}$ its transpose. Thus, the concurrence matrix for the i-th phase and j-th replicate is given by $\boldsymbol{N_{ij}}\boldsymbol{N_{ij}^{T}}$, which is summed over phases and replicates to get the concurrence matrix $\boldsymbol{M}$. The diagonal of $\boldsymbol{M}$ equals rp = 8 and the off-diagonal elements (λ_{hk}) equal the number of concurrences of a specific treatment comparison (treatments h and k), *i.e.* the number of times the hk-th treatment pair occurred in the same block. As $\boldsymbol{M}$ is symmetric only the upper off-diagonal elements were considered.

2 Two-phase designs with the same block structure in both phases

Corresponding to two-phase designs implemented in *Scenarios I* to *V*, the number of concurrences were counted from designs with r = 4 replicates, each having b = 84 incomplete blocks of size k = 8 in each phase.

Given the design parameters, 1260 direct comparisons of genotypes were expected in incomplete blocks of one replicate within the two phases. Hence, a maximum of 10080 direct comparisons across the two phases were possible. Because of the large treatment number and the small incomplete block size, a large number treatment pairs will never occur together in a block (λ_{hk} = 0 in Table A1 and Table A3). The number for λ_{hk} = 0 was smaller for *Scenarios III* and *IV* (approaches based on eight replicates or dummy coding to consider the both phases of the design) in comparison to the other scenarios, because *Scenarios III* and *IV* showed the maximum number of single concurrences (λ_{hk} = 1) across phases.

Further, in the case of transmitting the experimental design from phase one to two (*Scenario I*), the number of concurrence was λ_{hk} = 2.

When for each phase a separate design was generated (*Scenario II*), mostly single concurrences were obtained. But approximately 190 treatment pairs occurred twice (λ_{hk} = 2) within incomplete blocks across phases.

The *Scenario V* was the only approach, for which one treatment pair occurred threefold (λ_{hk} = 3) in incomplete blocks across phases.

Table A1. Treatment concurrences across the two phases for designs with the same block structure in both phases implemented in *Scenarios I* to *V* generated under Option 1 or Option 2.

	Option 1		Option 2		
λ_{hk}†	*Scenario I*	*Scenario II*	***Scenario III***	***Scenario IV***	*Scenario V*
0	121968	117119	**116928**	**116928**	117207
1	0	9698	**10080**	**10080**	9523
2	5040	191	**0**	**0**	277
3	0	0	**0**	**0**	1

†Equals the number of concurrences of a specific treatment comparison (treatments h and k) across incomplete blocks in phase one and two

Changing the variance components

To explore sensitivity of the ranking of designs in terms of $MVD_{(R)}$, we changed the values of variance components for block effects to different degrees to obtain the $MVD_{(R)}$ using the inter-block-intra-block dummy analysis (Table A2).

The ranks of *Scenarios I* to *V* remained the same and relevant differences between scenarios in $MVD_{(R)}$ were not obtained, except for *Scenario I*, which is in accordance to our findings of the main text.

Table A2. The $MVD_{(R)}$ obtained for two different sets of variance components of block effects assuming in each phase the same block structure.

	Variance component	
	Value set 1	Value set 2
REP	1	5
REP.IB1	2	1
REP.IB2	2	4
Error	3	2
	MVD	
Scenario I	**1.84012**	**1.24744**
Scenario II	2.16149	1.4871
Scenario III	2.15961	1.4855
Scenario IV	2.15981	1.48575
Scenario V	2.17225	1.4961

3 Two-phase designs with different block structures in both phases

Corresponding to two-phase designs implemented in *Scenario VI, VIII* to *X*, the number of treatment concurrences were counted from designs with a row-column design in the first phase and an IBD in the second phase. First, concurrences were obtained across rows and incomplete blocks in phase one and two. Secondly, concurrences were obtained across columns and incomplete blocks of both phases.

It is noted that the rows had the same size as incomplete blocks of the previous scenarios (Section 2) and hence the same maximum number (10080) of single concurrences could be expected across rows and incomplete blocks across phases (Table A3). The number of

concurrences across columns and incomplete blocks of phase one and two was much higher, as one column comprised 84 genotypes.

The two-phase design generated by the use of phase specific dummy variables (*Scenario VIII*) showed the maximum number single concurrences (λ_{hk} = 1) across rows and incomplete blocks (Table A3). Further, most treatment pairs occurred once (λ_{hk} = 1) across columns and incomplete blocks in phase one and two, whereas concurrences of 2, 3 and 4 were rare.

Table A3. Treatment concurrences across the two phases for Scenarios considering a row-column design in P1 and a resolvable IBD in P2 generated under Option 1 or Option 2.

λ_{hk}†	Option 1		Option 2			
	Scenario VI		***Scenario VIII***		*Scenario X*	
	ROW-IB	COL-IB	**ROW-IB**	**COL-IB**	ROW-IB	COL-IB
0	117146	59038	**116928**	**57668**	117202	58995
1	9644	49692	**10080**	**52015**	9534	49788
2	218	15955	**0**	**15357**	270	15889
3	0	2191	**0**	**1897**	2	2206
4	0	131	**0**	**71**	0	130
5	0	1	**0**	**0**	0	0

†Equals the number of concurrences of a specific treatment comparison (treatments h and k) either across rows and incomplete blocks or across columns and incomplete blocks in phase one and two

Changing the variance components

We changed the values of variance components for block effects to different degrees to obtain the $MVD_{(R)}$ using the inter-block-intra-block dummy analysis (Table A3).

The ranks of scenarios according to the $MVD_{(R)}$ remained unchanged compared to the ranking shown in the main text.

Table A3. The $MVD_{(R)}$ obtained for four different sets of variance components of block effects assuming in both phase a different block structure.

	Variance components			
	Value set 1	Value set 2	Value set 3	Value set 4
REP	6	1	1	1
REP.ROW	3	5	2	2
REP.COL	2	5	5	0.5
REP.IB2	4	1	1	5
Error	1	1	1	6
	MVD			
Scenario VI	0.8457	0.80775	0.78927	4.23265
Scenario VIII†	**0.84083**	**0.80478**	**0.78652**	**4.22416**
Scenario X	0.8555	0.81745	0.79795	4.26067

†The approach using a dummy variable to consider the both phases of the two-phase design
