# Supplementary material for: Identifying Effective Design Approaches to Allocate Genotypes in Two-Phase Designs: A Case Study in Pelargonium zonale
Source: Front Plant Sci. 2018 Jan 5;8:2194. doi: 10.3389/fpls.2017.02194 (PMC5760546; doi:10.3389/fpls.2017.02194)
Supplement: PRESENTATION 4 — Concurrence of two-phase designs. [file Presentation_4.PDF]

Scenario I – The same design in both phases (the same genotypes in the same blocks across phases)

Replicate 1

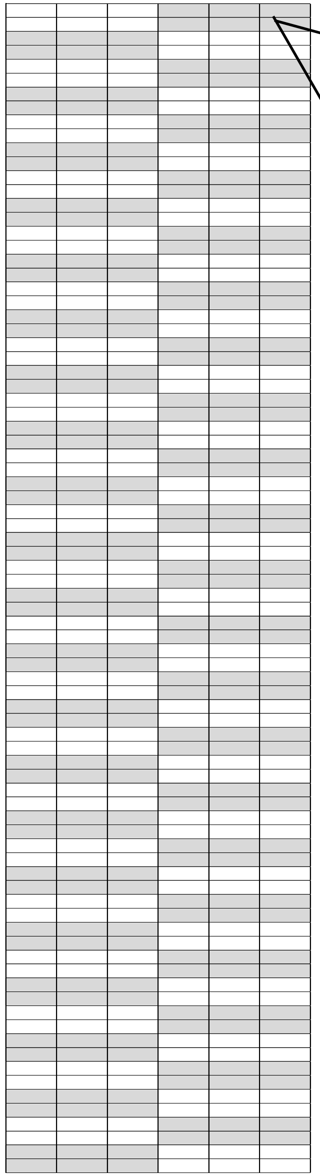

Phase 1, replicate 1, block 84

134	175	378
164	355	468

Phase 2, replicate 1, block 84

134	175	378
164	355	468

In both phases, exactly the same experimental layout was considered by transmitting the experimental layout (Code 1) from phase one to phase two.

Replicate 1 represents the first replicate in both phases in Scenario I.
As the same layout was considered in both phases, the same genotypes were tested on the same experimental units in phase one and two. This is indicated by the same genotypes in block 84 of the first replicate in both phases.

Figure A

Scenario II – In each phase a randomization took place (genotypes in different blocks across phases)

Replicate 1

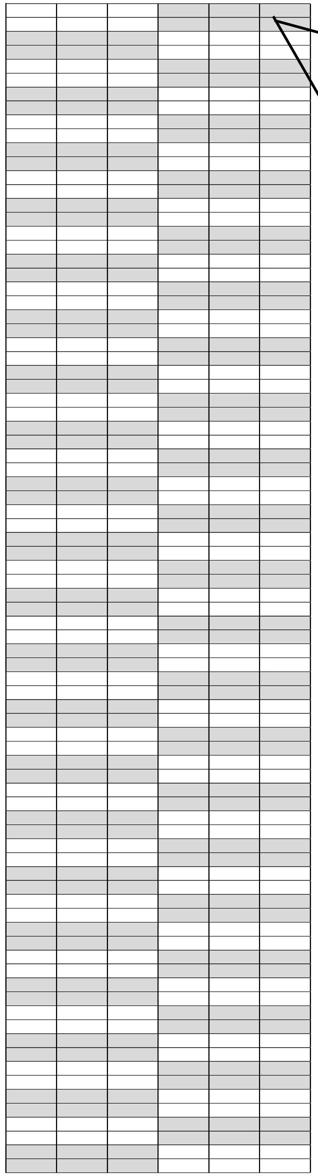

Phase 1, replicate 1, block 84

129	71	497
462	170	437

Phase 2, replicate 1, block 84

224	261	90
122	256	25

In both phases, the same block structure was considered, however, the experimental layout was generated for each phase separately (Code 2).

Replicate 1 represents the first replicate of both designs. As two designs were generated, different genotypes were tested in the last block of the first replicate .
This is indicated by the different genotypes in entries in block 84.

Figure B

Scenario III – A randomization across 8 replicates assigning the first 4 to phase 1, the other to phase 2

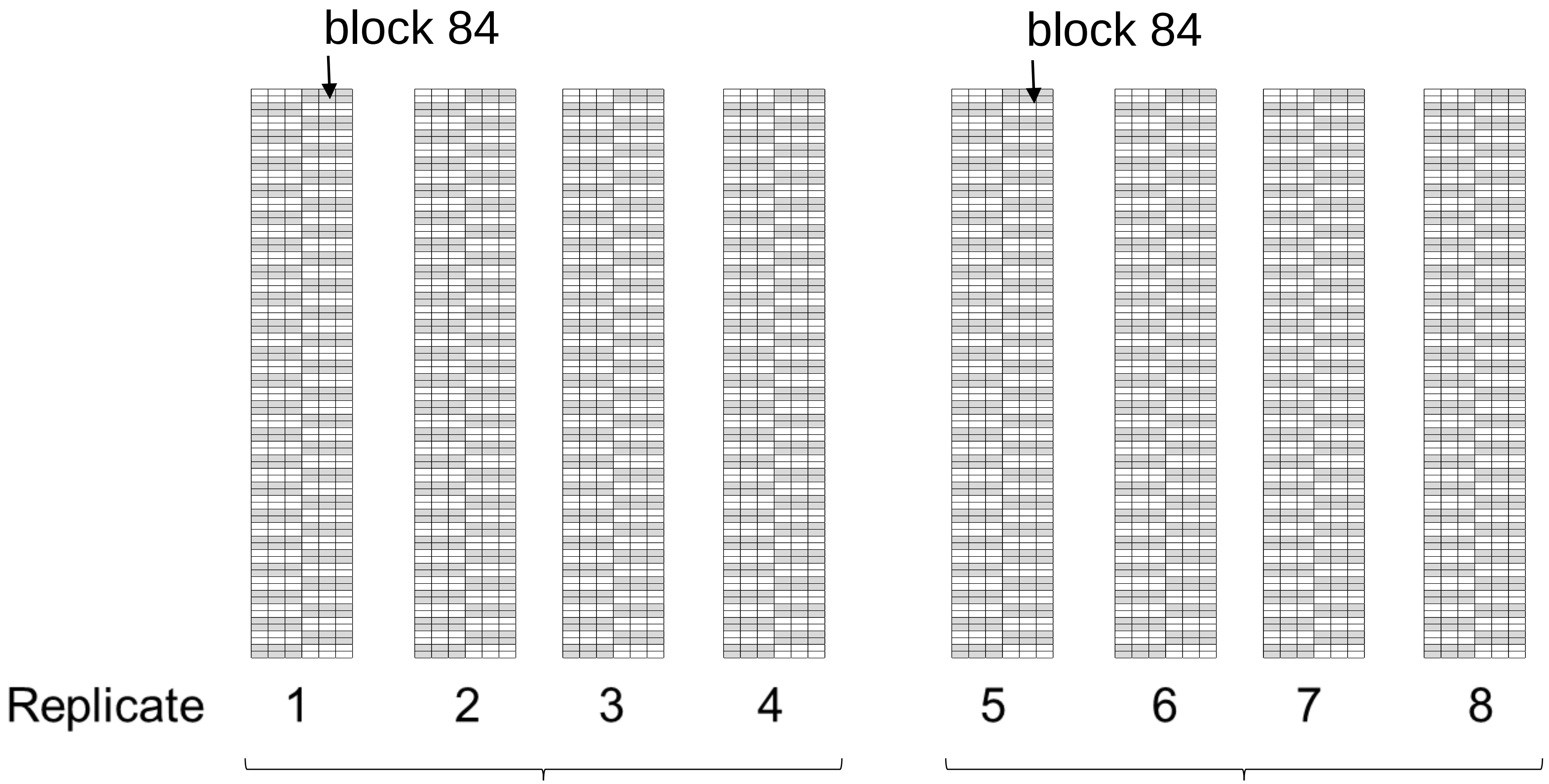

To generate a two-phase design across phases, each having the same block structure, the replicate number was increased to 8 (Code 3). The first four replicates were assigned to phase one, the remaining four were assigned to phase two.
Thus, different genotypes were tested in block 84 of the first replicate, in each phase.

451	461	159
178	105	434

Phase 1, replicate 1 block 84

38	64	436
68	395	23

Phase 2, replicate 5, block 84

Figure C

Scenario IV – Separation of the block structure by dummy coding

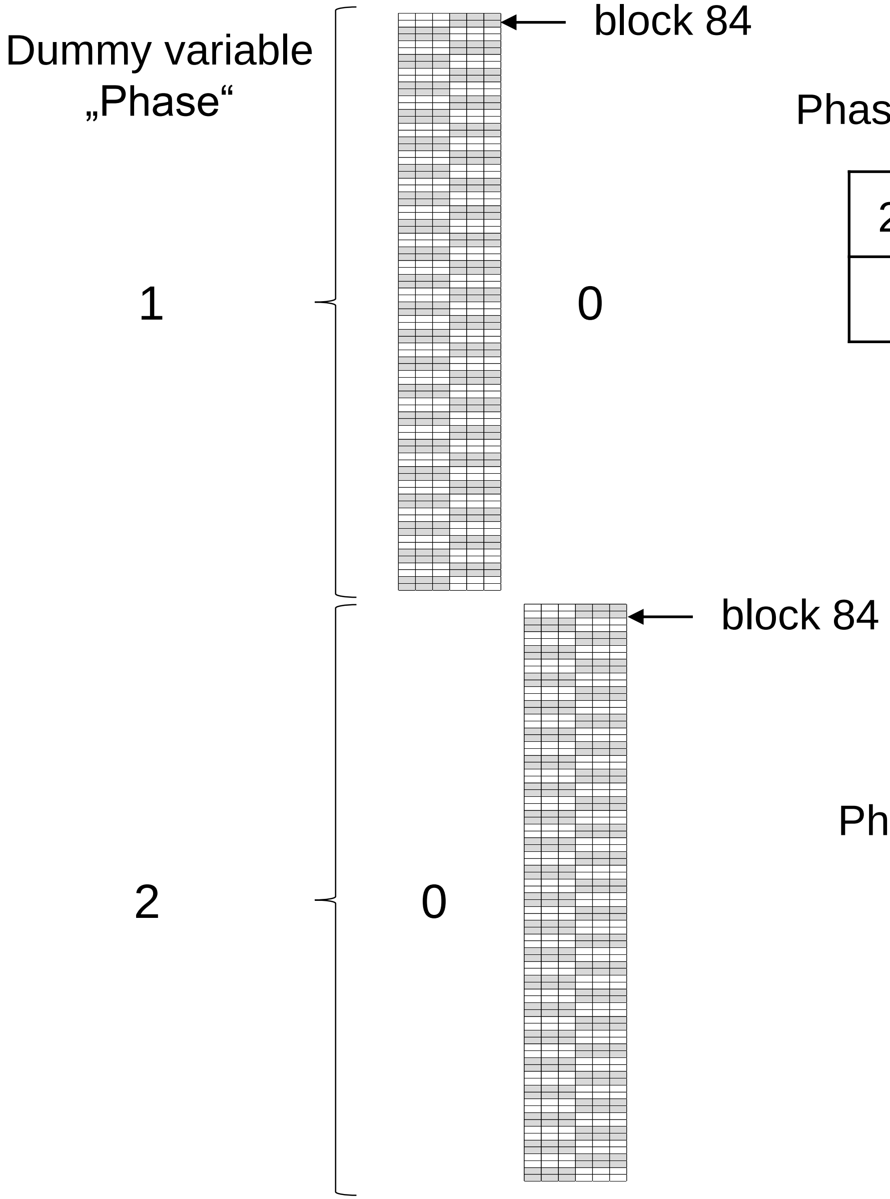

Phase 1, replicate 1 block 84

274	312	282
90	309	177

Phase 2, replicate 1 block 84

113	2	136
392	37	40

In each phase, there were *r* = 4 replicates and incomplete blocks were nested within each of the replicates. The records for the two phases were concatenated in the dataset for design generation. The clue for generating the design across the two phases was to set the factor for incomplete blocks of P1 to a single pseudo level for incomplete blocks of P2 and vice versa. By this dummy coding, the pseudo level acted as one additional block level of incomplete blocks in P1 or P2. The design was then optimized simultaneously with respect to the assignment of genotypes to the two blocking systems.

Figure D

Scenario V – Randomization in replicate-wise order

Step I: Allocating blocks of P2 to blocks in P1

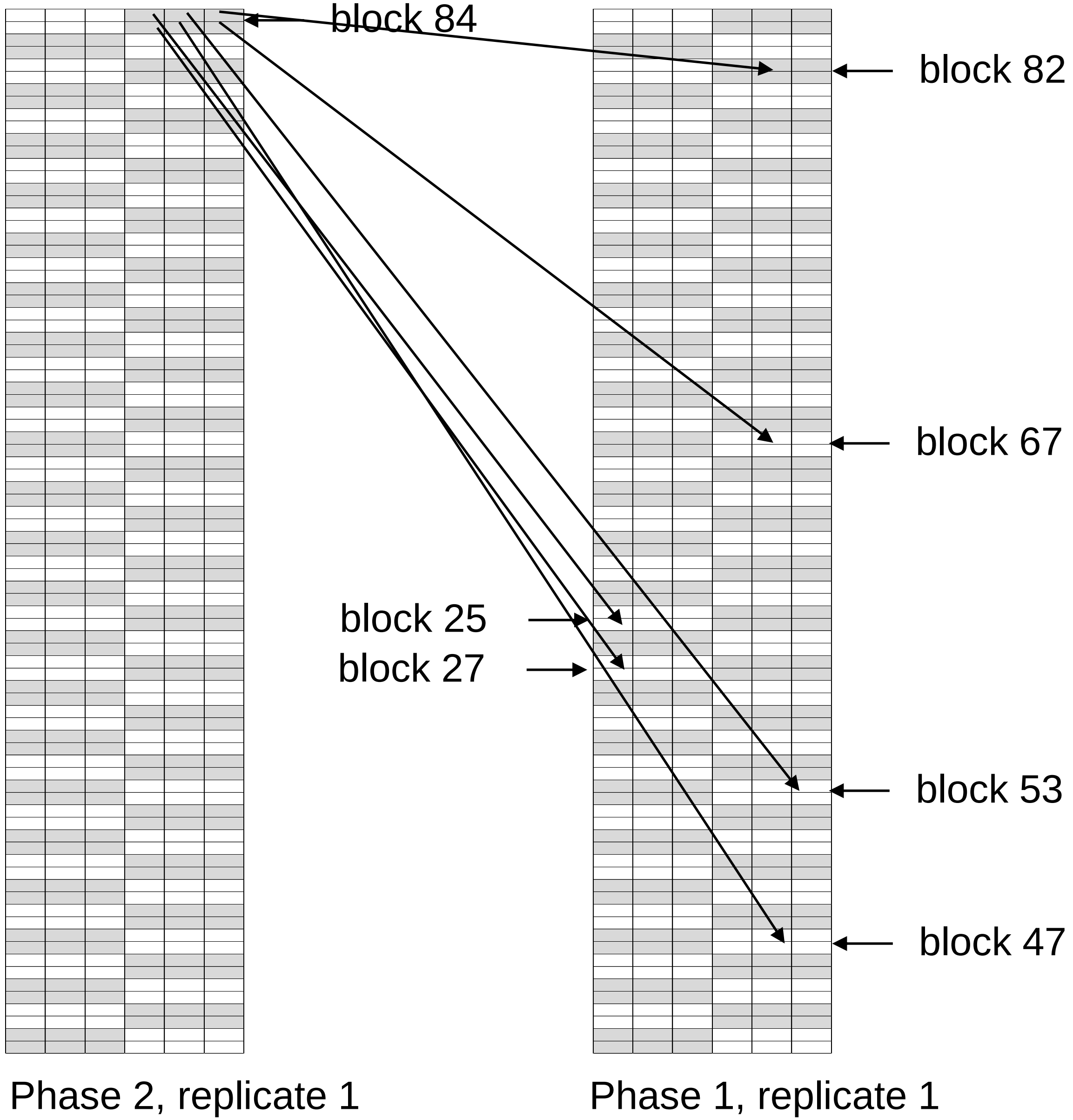

To generate a design across phases in replicate-wise order, allocate the blocks of phase 2 to blocks in phase 1 (Code 5), i.e. the block entries of the second phase were randomly allocated to other blocks in the first phase.

Figure E1

Scenario V – Randomization in replicate-wise order

Step II: Allocating genotypes to blocks in P1 and P2

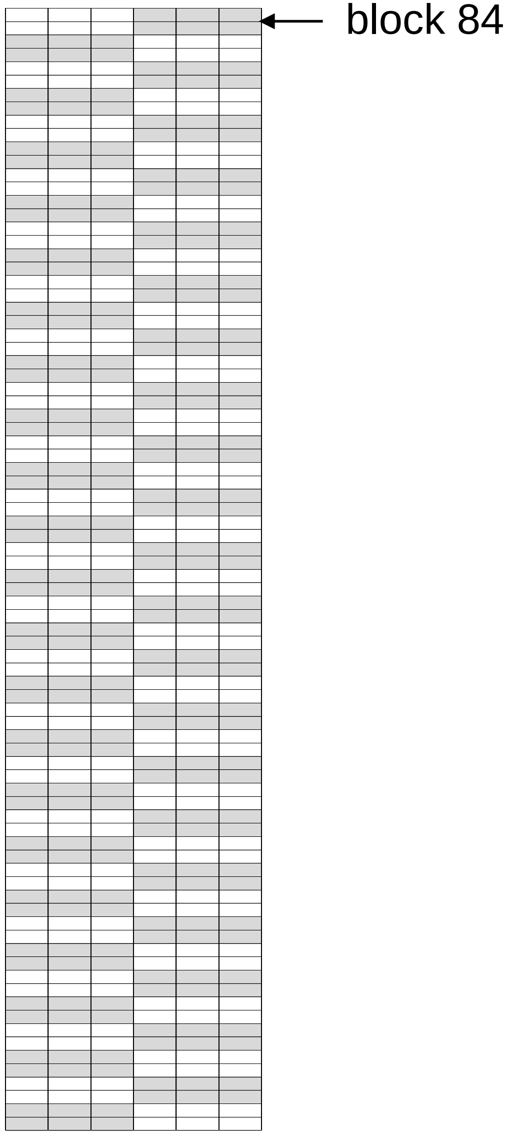

Phase 1, replicate 1

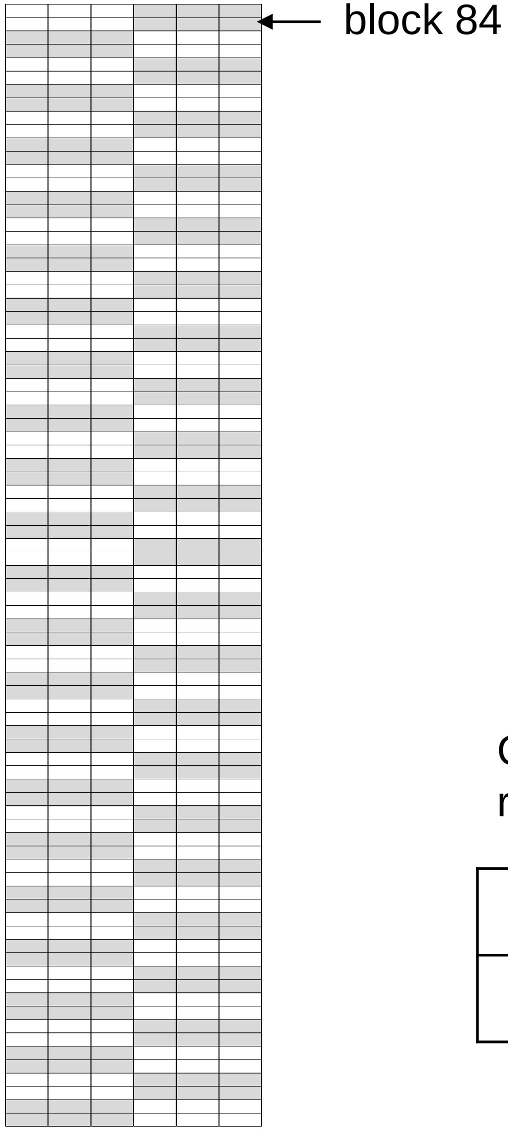

Phase 1, replicate 2

To generate a design across phases in replicate-wise order, allocate the genotypes to blocks of phase 1 and 2 simultanously (Code 5).
Thus, different genotypes were tested in block 84 of the first replicate, in each phase.

Genotypes, phase 1, replicate 1, block 84

283	325	213
222	268	64

Genotypes, phase 2, replicate 1, block 84

267	63	195
273	329	258

Figure E2
